# Effect of the 2-R-Allyl and Chloride Ligands on the Cathodic Paths of [Mo(η^3^-2-R-allyl)(α-diimine)(CO)_2_Cl] (R = H, CH_3_; α-diimine = 6,6′-Dimethyl-2,2′-bipyridine, Bis(*p*-tolylimino)acenaphthene)

**DOI:** 10.1021/acs.organomet.1c00038

**Published:** 2021-06-02

**Authors:** James O. Taylor, Ryan Culpeck, Ann M. Chippindale, Maria José Calhorda, František Hartl

**Affiliations:** † Department of Chemistry, University of Reading, Reading RG6 6DX, United Kingdom; ‡ BioISI-Biosystems & Integrative Sciences Institute, Departamento de Química e Bioquímica, Faculdade de Ciências, Universidade de Lisboa, 1749-016 Lisbon, Portugal

## Abstract

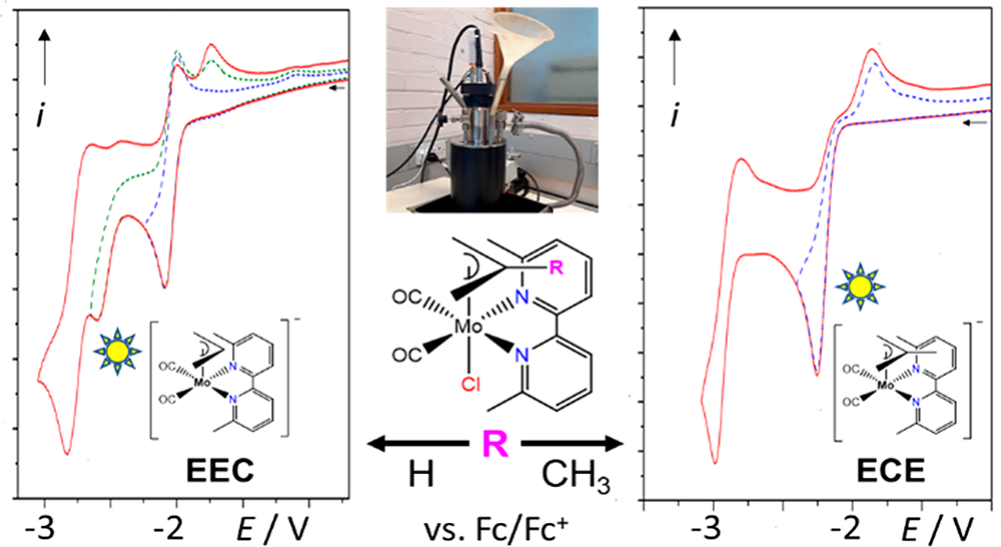

The new, formally Mo(II) complexes [Mo(η^3^-2-R-allyl)(6,6′-dmbipy)(CO)_2_Cl] (6,6′-dmbipy = 6,6′-dimethyl-2,2′-bipyridine; 2-R-allyl = allyl for R = H, 2-methallyl for R = CH_3_) and [Mo(η^3^-2-methallyl)(pTol-bian)(CO)_2_Cl] (pTol-bian = bis(p-tolylimino)acenaphthene) share, in this rare case, the same structural type. The effect of the anionic π-donor ligand X (Cl^–^ vs NCS^–^) and the 2-R-allyl substituents on the cathodic behavior was explored. Both ligands play a significant role at all stages of the reduction path. While 2e^–^-reduced [Mo(η^3^-allyl)(6,6′-dmbipy)(CO)_2_]^−^ is inert when it is ECE-generated from [Mo(η^3^-allyl)(6,6′-dmbipy)(CO)_2_(NCS)], the Cl^–^ ligand promotes Mo–Mo dimerization by facilitating the nucleophilic attack of [Mo(η^3^-allyl)(6,6′-dmbipy)(CO)_2_]^−^ at the parent complex at ambient temperature. The replacement of the allyl ligand by 2-methallyl has a similar effect. The Cl^–^/2-methallyl ligand assembly destabilizes even primary radical anions of the complex containing the strongly π-accepting pTol-Bian ligand. Under argon, the cathodic paths of [Mo(η^3^-2-R-allyl)(6,6′-dmbipy)(CO)_2_Cl] terminate at ambient temperature with 5-coordinate [Mo(6,6′-dmbipy)(CO)_3_]^2–^ instead of [Mo(η^3^-2-R-allyl)(6,6′-dmbipy)(CO)_2_]^−^, which is stabilized in chilled electrolyte. [Mo(η^3^-allyl)(6,6′-dmbipy)(CO)_2_]^−^ catalyzes CO_2_ reduction only when it is generated at the second cathodic wave of the parent complex, while [Mo(η^3^-2-methallyl)(6,6′-dmbipy)(CO)_2_]^−^ is already moderately active at the first cathodic wave. This behavior is fully consistent with absent dimerization under argon on the cyclic voltammetric time scale. The electrocatalytic generation of CO and formate is hampered by the irreversible formation of anionic tricarbonyl complexes replacing reactive [Mo(η^3^-2-methallyl)(6,6′-dmbipy)(CO)_2_]_2_ along the cathodic route.

## Introduction

There is a strong interest in the electrocatalytic reduction of CO_2_ that offers a sustainable route to a variety of valuable chemical feedstocks for organic synthesis or chemical fuel. Transition-metal complexes have been identified as highly effective catalysts for the 2e^–^ reduction of CO_2_, allowing one to take advantage of energy-saving proton-coupled pathways.^1,2^ The original reports have mostly focused on complexes based on rare and precious metals, such as rhenium in [Re(bipy)(CO)_3_Cl] (bipy = 2,2′-bipyridine), where the active catalyst is the 2e^–^-reduced 5-coordinate anion [Re(bipy)(CO)_3_]^−^.^3−7^ The costs associated with such materials directed current research efforts toward Earth-abundant metals, such as Mn. [Mn(bipy)(CO)_3_]^−^ has only recently been identified as a catalyst in the presence of small amounts of Brønsted acids.^8−11^ Although catalysts with impressive performance based on Earth-abundant first-row transition metals, such as Fe, Co and Ni, are now widely known,^12^ much less attention has been paid to the Group 6 metals (Cr, Mo, W).

Currently, the limited literature dealing with the Group 6 metals has largely addressed two families of complexes: viz., [Mo(α-diimine)(CO)_4_]^13−19^ and [Mo(η^3^-allyl)(α-diimine)(CO)_2_X] (X = halide, pseudohalide).^20,21^ The hexacarbonyl precursor, [Mo(CO)_6_], is also active toward the 2e^–^ electrocatalytic reduction of CO_2_, unlike the equivalent Group 7 complexes [M(CO)_5_]_2_ and [M(CO)_5_X].^22^ The complexes [Mo(α-diimine)(CO)_4_] (α-diimine = 2,2′-bipyridine or *x,x′*-dimethylbipyridine (*x* = 4–6)) undergo reversible reduction to [Mo(α-diimine)(CO)_4_]^•–^ and subsequent reduction to the 6-coordinate transient [Mo(α-diimine)(CO)_4_]^2–^, converting concomitantly to the 5-coordinate catalyst [Mo(α-diimine)(CO)_3_]^2–^. The onset of the catalytic wave may be shifted to less negative potentials, due to an equilibrium between [Mo(α-diimine)(CO)_4_]^•–^ and [Mo(α-diimine)(CO)_3_]^•–^ at an Au cathodic surface facilitating CO dissociation from the usually stable tetracarbonyl radical anion. The transient 5-coordinate radical anion is reducible to the active dianionic catalyst at ca. 500 mV less negative overpotentials. Smart choices of solvent and electrode materials, coupled with ligand effects, make this class of metal catalysts more comparable in CO_2_ electroreduction performance with those of other Earth-abundant metals.^21^ This process can further be enhanced by photoassisted activation of [Mo(α-diimine)(CO)_4_]^•–^.^23^

**Scheme 1:**
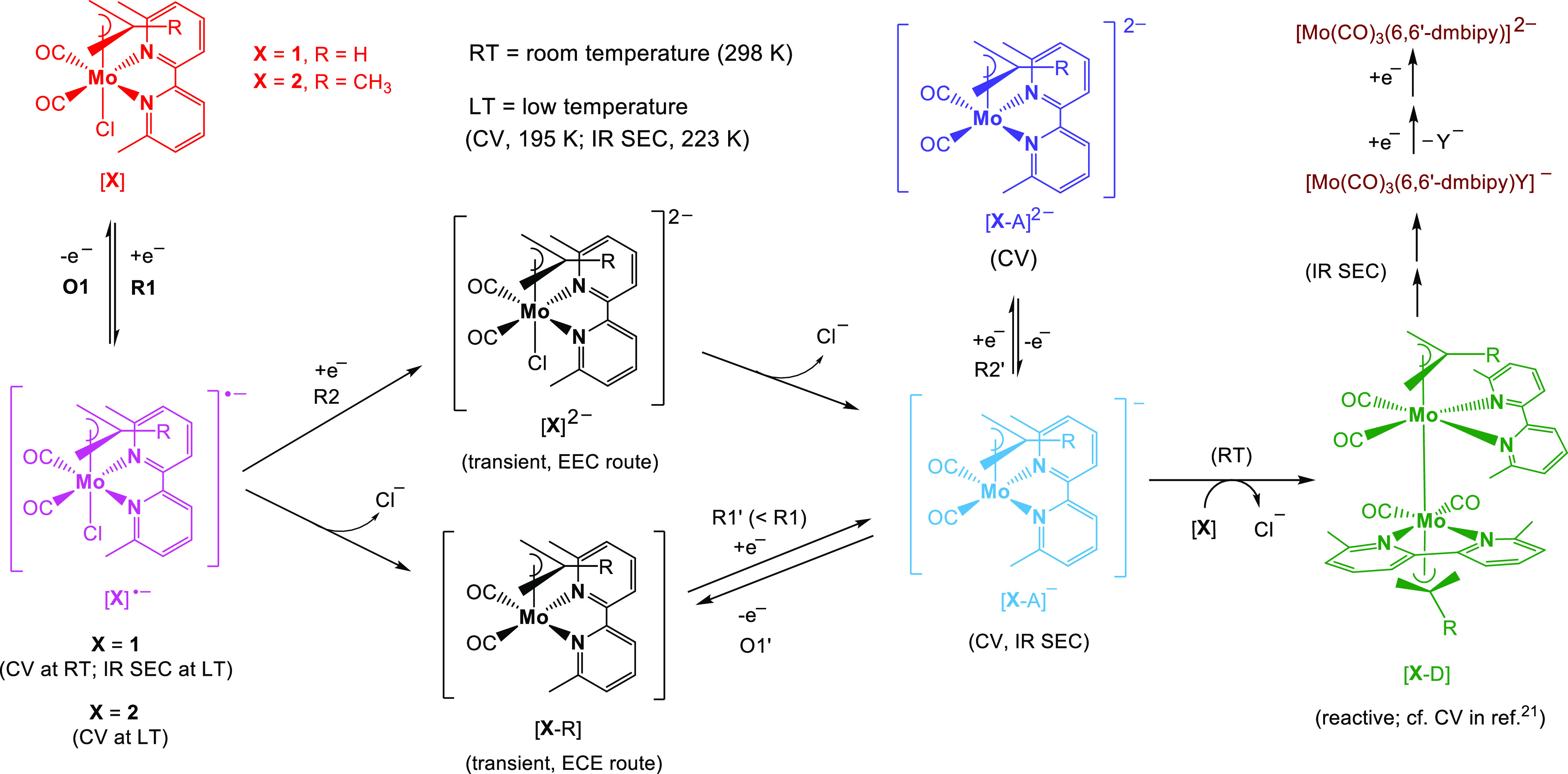
Cathodic Pathways of the Complexes [Mo(η^3^-allyl)(6,6′-dmbipy)(CO)_2_Cl] (**1**) and [Mo(η^3^-2-methallyl)(6,6′-dmbipy)(CO)_2_Cl] (**2**), on the Basis of the Evidence from Cyclic Voltammetry (CV) and IR Spectroelectrochemistry (IR SEC)

The complexes in the second class, [Mo(η^3^-allyl)(α-diimine)(CO)_2_X] (α-diimine = 2,2′-bipyridine, *x,x′*-dimethylbipyridine (*x* = 4–6); X = halide, pseudohalide), have been identified as precursors to the catalytically active 5-coordinate anion [Mo(η^3^-allyl)(α-diimine)(CO)_2_]^−^.^20,21^ In contrast to the Group 6 tetracarbonyls introduced above, the parent complex [Mo(η^3^-allyl)(bipy)(CO)_2_(NCS)] is reduced irreversibly to an unstable radical anion, triggering the loss of the NCS^–^ ligand with concomitant reduction of the 5-coordinate radical to the 5-coordinate anion. A dimer, viz. [Mo(η^3^-allyl)(bipy)(CO)_2_]_2_, is formed under ambient conditions by a zero-electron coupling reaction of the 2e^–^-reduced 5-coordinate anion with the yet nonreduced parent complex, in a manner very similar to the ECEC reduction path of [Mn(bipy)(CO)_3_Br], leading to [Mn(bipy)(CO)_3_]_2_.^8,24−26^ In contrast to the latter dimer, the Mo(allyl)-based dimer is quite reactive and could not be reduced to the corresponding 5-coordinate anion.^20,21^ In the subsequent study of [Mo(η^3^-allyl)(*x,x′*-dmbipy)(CO)_2_(NCS)] (*x* = 4–6), it was revealed what factors control the persistence of the 5-coordinate anion,^21^ since the complexes are quite susceptible to electronic and steric changes in the ligand coordination sphere. For instance, the primary 1e^–^-reduced radical anion, [Mo(η^3^-allyl)(6,6′-dmbipy)(CO)_2_(NCS)]^•–^, was stable at room temperature on the CV time scale, radically altering the reduction pathway from ECEC to EEC (resembling more the reduction path of [Mo(bipy)(CO)_4_]). This allowed the active 5-coordinate anionic catalyst, [Mo(η^3^-allyl)(6,6′-dmbipy)(CO)_2_]^−^, to be characterized for the first time by IR spectroelectrochemistry. The molecular structures of the transient, intermediate, and ultimate reduced species are visualized in Scheme 1 in ref (21) and the new Scheme 1 presented here.

**Chart 1:**
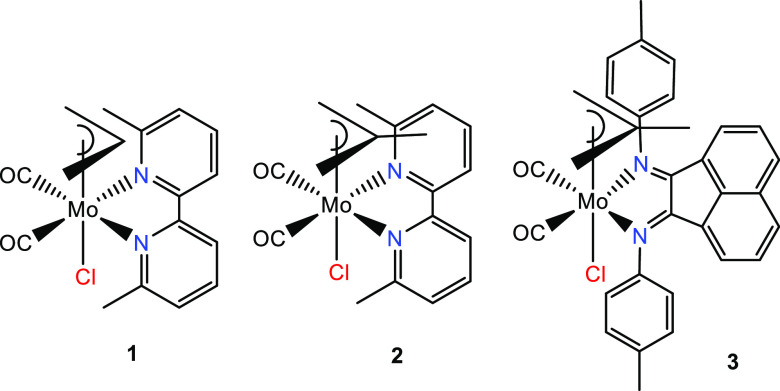
Molecular Structures of the Studied Complexes, [Mo(η^3^-allyl)(6,6′-dmbipy)(CO)_2_Cl] (**1**), [Mo(η^3^-2-methallyl)(6,6′-dmbipy)(CO)_2_Cl] (**2**), and [Mo(η^3^-2-methallyl)(pTol-Bian)(CO)_2_Cl] (**3**)

The present work aims at complementing the valuable insight into the cathodic paths of these Mo(II) complexes, gathered from the [Mo(η^3^-allyl)(*x,x′*-dmbipy)(CO)_2_(NCS)] (*x* = 4–6) series,^21^ with new mechanistic details. The first complex in the new group, [Mo(η^3^-allyl)(6,6′-dmbipy)(CO)_2_Cl] (**1** in Chart 1), probes the effect of changing the anionic X^–^ ligand from a moderate π-donor ligand, NCS^–^, to the stronger σ- and π-donor, Cl^–^. This substitution affects the stability of the singly reduced species and controls the reactivity of the parent complex toward the ECEC dimerization coupling. The second complex, [Mo(η^3^-2-methallyl)(6,6′-dimethyl-2,2′-bipyridine)(CO)_2_Cl] (**2** in Chart 1), allows the effect of the allylic methyl substitution at the meso-C atom on the cathodic steps to be investigated and compared with the methyl substitution at the pyridyl rings of the equatorial 2,2′-bipyridine ligand.

Finally, a third complex, [Mo(η^3^-2-methallyl)(pTol-Bian)(CO)_2_Cl] (**3** in Chart 1; pTol-Bian = bis(*p*-tolylimino)acenaphthene), unusually exhibiting the same structural type as **1** and **2**, but having an extended conjugated *N*-aryl-Bian π-system, was prepared as a reference compound, featuring a strong π-acceptor ligand, in contrast to the 6,6′-dmbipy counterpart.

Thus, the ultimate goals of the study were to probe (i) the steric and electronic consequences of allylic methyl substitution on the cathodic path, (ii) the effect of the Cl^–^ ligand in comparison to SCN^–^ on the structures and reactivity of the reduced complexes, and (iii) the effect of the alternative coordination sphere including a stronger π-acceptor redox-active ligand. At the same time, the peculiar secondary reactivity accompanying the dimerization step along the cathodic path at ambient temperature was further explored to assign the ultimate reduction products.

## Experimental Section

### Materials and Methods

All synthetic and electrochemical operations were carried out under an atmosphere of dry argon gas using standard Schlenk techniques. Tetrahydrofuran (THF) was freshly distilled under dry argon from ketyl radicals derived from the reaction of metallic Na and benzophenone, butyronitrile (PrCN) and dichloromethane (DCM) were distilled from CaH_2_, and acetonitrile (MeCN) was distilled from P_2_O_5_. The supporting electrolyte, Bu_4_NPF_6_ (Acros Organics), was recrystallized twice from ethanol and dried under vacuum at 373 K for 5 h. Just prior to the experiment, the electrolyte was dried again overnight at 373 K. The precursor complexes, [Mo(η^3^-2-R-allyl)(MeCN)_2_(CO)_2_Cl] (R = H, CH_3_), were prepared in good yields by a slight modification of the literature procedures.^27^ The ligand pTol-Bian was prepared according to a literature procedure involving the condensation reaction of acenaphthenequinone and 2,6-dimethylaniline.^28^ All other compounds were purchased from Sigma-Aldrich and used without further purification. The target complexes were prepared by facile thermal substitution of the labile MeCN ligands in the precursor complexes. The purity and identity of the final products were confirmed by infrared and NMR spectroscopy and single-crystal X-ray diffraction. ^1^H NMR spectra were recorded on a 400 MHz Bruker NanoBay spectrometer. Elemental analyses were carried out by Medac Ltd.

### General Synthesis of [Mo(η^3^-2-R-allyl)(α-diimine)(CO)_2_Cl] (R = H (1), CH_3_ (2))

A solution of [Mo(η^3^-2-R-allyl)(MeCN)_2_(CO)_2_Cl] (0.62 mmol, 0.2 g) in dry DCM (10 mL) was mixed under dry argon with a solution of the appropriate α-diimine ligand (typically 0.65 mmol) in dry DCM (10 mL). The mixture was stirred for 4 h, and then the volume was reduced by half. The crude product was precipitated by slow addition of hexane (10 × 5 mL). Roughly 100 mg of the precipitate was recovered by inert filtration and washed with cold hexane (2 × 10 mL). Spectroscopically pure samples were prepared by column chromatography on silica, using either DCM/hexane (9/1, v/v) or DCM/diethyl ether (9/1, v/v) as the eluent, where necessary. Following the purification, yields ranged between 15 and 50%. Crystals for X-ray analysis were grown by slow evaporation of DCM.

### [Mo(η^3^-allyl)(6,6′-dimethyl-2,2′-bipyridine)(CO)_2_Cl] (**1**)

The precursor [Mo(η^3^-allyl)(MeCN)_2_(CO)_2_Cl] (0.62 mmol, 200 mg) reacted with 6,6′-dimethyl-2,2′-bipyridine (0.54 mmol, 100 mg) to afford complex **1**. The crude product was purified on a silica column, using DCM/hexane (9/1, v/v) as the eluent. Yield: 30 mg, 15%. ^1^H NMR (400 MHz, DCM-*d*_2_): δ 7.83 (2H, d, *J* = 8 Hz), 7.75 (2H, t, *J* = 8 Hz), 7.32 (2H, d, *J* = 8 Hz), 2.95 (6H, s), 2.66 (1H, m), 2.49 (2H, d, *J* = 8 Hz), 1.05 (2H, d, *J* = 8 Hz). IR (ν(CO), THF): 1945, 1861 cm^–1^. Anal. Calcd for C_17_H_17_ClMoN_2_O_2_(CH_2_Cl_2_) (497.64): C, 43.44; H, 3.84; N, 5.62. Found: C, 43.56; H, 3.99; N, 5.38.

### [Mo(η^3^-2-methallyl)(6,6′-dimethyl-2,2′-bipyridine)(CO)_2_Cl] (**2**)

The precursor [Mo(η^3^-2-methallyl)(MeCN)_2_(CO)_2_Cl] (0.62 mmol, 200 mg) reacted with 6,6′-dimethyl-2,2′-bipyridine (0.54 mmol, 100 mg), to afford complex **2**. The crude product was filtered and washed with cold hexane (2 × 10 mL). Yield: 97 mg, 42%. ^1^H NMR (400 MHz, DCM-*d*_2_): δ 7.87 (2H, d, *J* = 8 Hz), 7.75 (2H, t, *J* = 8 Hz), 7.34 (2H, d, *J* = 8 Hz), 2.95 (6H, s), 2.34 (2H, s), 1.18 (2H, s), 0.98 (3H, s). IR (ν(CO), THF): 1944, 1861 cm^–1^. Anal. Calcd for C_18_H_19_ClMoN_2_O_2_ (426.75): C, 50.66; H, 4.48; N, 6.56. Found: C, 50.42; H, 4.40; N, 6.52.

### [Mo(η^3^-2-methallyl)(bis(*p*-tolylimino)acenaphthene)(CO)_2_Cl] (**3**)

The precursor [Mo(η^3^-2-methallyl)(MeCN)_2_(CO)_2_Cl] (0.62 mmol, 200 mg) reacted with bis(*p*-tolylimino)acenaphthene (0.54 mmol, 195 mg) to afford complex **3**. The crude product was purified on a silica column, using DCM/diethyl ether (9/1, v/v) as the eluent. Yield: (45 mg, 15%). ^1^H NMR (400 MHz, DCM-*d*_2_): δ 7.87 (2H, d, *J* = 8 Hz), 7.58 (2H, d, *J* = 8 Hz), 7.32 (6H, m), 7.01 (2H, d, *J* = 8 Hz), 6.78 (2H, d, *J* = 8 Hz) 2.63 (2H, s), 2.03 (3H, s), 1.18 (2H, s). IR (ν(CO), THF): 1956, 1886 cm^–1^. Anal. Calcd for C_32_H_27_ClMoN_2_O_2_ (602.97): C, 63.74; H, 4.17; N, 5.80%. Found: C, 63.61; H, 4.51; N, 5.67%.

### X-ray Structure Determination

Crystals were mounted under Paratone-N oil and flash-cooled to either 100 K (for **1·**CH_2_Cl_2_ and **3**) or 200 K (for **2**) in a stream of nitrogen in an Oxford Cryostream 800 cooler. Single-crystal X-ray intensity data were collected using a Rigaku XtaLAB Synergy diffractometer (Cu Kα radiation (λ = 1.54184 Å)). The data were reduced within the CrysAlisPro software.^29^ The structures were solved using the program Superflip,^30^ and all non-hydrogen atoms were located. Least-squares refinements were performed using the CRYSTALS suite of programs.^31^ The non-hydrogen atoms were refined anisotropically. Each hydrogen atom on the ligands was placed geometrically at a C–H distance of 0.95 Å with a *U*_iso_ value of 1.2–1.5 times the *U*_eq_ value of the parent C atom. The positions of the hydrogen atoms were then refined with riding constraints. CCDC codes: 1989618 for **1·**CH_2_Cl_2_, 1989622 for **2**, and 1989623 for **3**.

### Cyclic Voltammetry

Cyclic voltammograms of complexes **1**–**3** were recorded with a Metrohm Autolab PGSTAT302N potentiostat operated with the NOVA 2.14 software. The airtight single-compartment electrochemical cell housed a Pt-microdisk working electrode (active area of 0.4 mm^2^) polished with 0.25 μm diamond paste (Kemet), a coiled-Pt-wire counter electrode, and a coiled-Ag-wire pseudoreference electrode. All values are reported vs the ferrocene/ferrocenium (Fc/Fc^+^) redox couple, which served as the internal standard for most measurements and was added just before the final potential sweep. Where necessary, decamethylferrocene (Fc*/Fc*^+^) served this purpose in order to avoid overlap with the nearby Mo(II)/Mo(III) oxidation. In THF, the value of *E*_1/2_(Fc*/Fc*^+^) = −0.48 V vs Fc/Fc^+^ has been determined for this work. Solutions contained 10^–1^ M Bu_4_NPF_6_ and 10^–3^ M analyte.

### IR Spectroelectrochemistry

IR spectroelectrochemical experiments were performed using a Bruker Vertex 70v FT-IR spectrometer. An internal DLaTGS detector and an external Bio-RAD FTS 60 MCT detector (linked to the spectrometer and housing the cryostat) served for measurements at *T* = 298 and 223 K, respectively. The *in situ* electrolyses at ambient temperature were conducted using an airtight OTTLE cell.^32^ The cell was equipped with Pt-minigrid (32 wires/cm) working and auxiliary electrodes, an Ag-microwire pseudoreference electrode, and optically transparent CaF_2_ windows. The course of the spectroelectrochemical experiment was monitored by thin-layer cyclic voltammetry. The electrode potential control during the thin-layer CV was achieved using a PalmSens EmStat3 potentiostat, operated with PSTrace5 software. Low-temperature spectroelectrochemical measurements were carried out with a cryostatted OTTLE cell of a similar design.^33^ Solutions contained 3 × 10^–1^ M Bu_4_NPF_6_ and 3 × 10^–3^ M analyte.

### Computational Studies

Density functional theory (DFT) calculations^34^ were performed using the Amsterdam Density Functional (ADF) program.^35−37^ Geometries were optimized without symmetry constraints using the local density approximation (LDA) of the correlation energy (Vosko–Wilk–Nusair)^38^ and the generalized gradient approximation (Becke’s^39^ exchange and Perdew’s^40,41^ correlation functionals) with gradient correction. Unrestricted calculations were performed for open-shell complexes. Solvent (THF) was considered in all geometry optimizations and single-point calculations, using the COSMO approach implemented in ADF. Relativistic effects were treated with the ZORA approximation.^42^ Triple-ζ Slater-type orbitals (STOs) were used to describe all of the valence electrons of H, O, C, N, Cl, and Mo. A set of two polarization functions was added to H (single ζ 2s, 2p), O, C, N, and Cl (single ζ, 3d, 4f), and Mo (5d, 4f). Frequency calculations were performed to obtain the vibrational spectra and to check that intermediates were minima in the potential-energy surface. Three-dimensional representations of the structures and molecular orbitals were obtained with Chemcraft.^43^

## Results and Discussion

### Characterization and Crystal Structure Analysis

In THF, the IR spectra of complexes **1**–**3** exhibit two ν(CO) bands. For complexes **1** and **2**, these absorption bands are almost identical in terms of both the intensity pattern and wavenumbers: viz., 1945 and 1861 cm^–1^. In comparison with the reference, [Mo(η^3^-allyl)(6,6′-dmbipy)(CO)_2_(NCS)] (1948, 1866 cm^–1^), the absorption bands are slightly shifted to smaller wavenumbers, which reflects the increased π-back-donation experienced by the CO ligands upon the replacement of NCS^–^ by the stronger π-donor Cl^–^. Finally, in complex **3**, the ν(CO) bands are observed at 1956 and 1886 cm^–1^, reflecting the increased acceptance from the pTol-Bian ligand in comparison with 6,6′-dmbipy in complex **2**.

**Figure 1:**
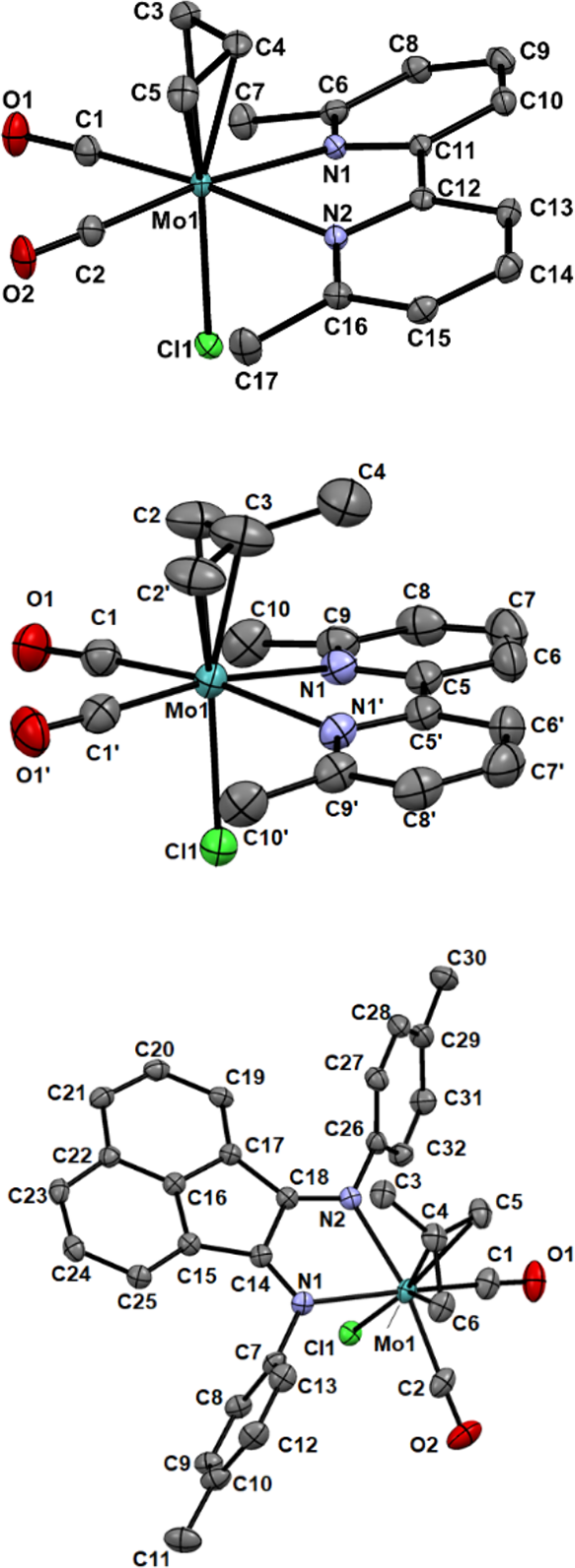
ORTEP views (50% thermal probability) of the molecular structures of [Mo(η^3^-allyl))(6,6′-dmbipy)(CO)_2_Cl] (**1·**CH_2_Cl_2_, top), [Mo(η^3^-2-methallyl)(6,6′-dmbipy)(CO)_2_Cl] (**2**, middle) and [Mo(η^3^-2-methallyl)(pTol-Bian)(CO)_2_Cl] (**3**, bottom) determined by single-crystal X-ray diffraction. Hydrogen atoms have been omitted for clarity. Symmetry code in **2**: (′) *x*, −*y* + 1/2, *z*.

The structures of **1·**CH_2_Cl_2_, **2**, and **3** are presented in Figure 1. Crystallographic data and selected bond lengths are summarized in Tables S1 and S2 in the Supporting Information. All three complexes adopt the type A pseudo-octahedral (equatorial) structure, which has been observed for the [Mo(η^3^-allyl)(*x*,*x*′-dmbipy)(CO)_2_(NCS)] (*x* = 4–6) series,^21^ with both donor nitrogen atoms of the chelating ligand N∩N in positions trans to the CO ligands. While most of the bipy complexes known exhibit this arrangement, it is worth noting that this structure is observed here for the first time in a complex of an *N*-aryl-Bian ligand. Indeed, all the analogous complexes with the large and strong π-acceptor, *N*-aryl-Bian, always adopt the less symmetrical type B (axial) structure.^20,44−48^ As has been widely observed previously, in all three complexes, the open face of the allyl ligand lies over the CO ligands (i.e., the *endo* isomer is preferred).

The Mo–Cl bond lengths in the three complexes show a trend reflecting subtle variations of the chloride environment: **2** (2.5145(15) Å) > **1**·CH_2_Cl_2_ (2.4914(8) Å) > **3** (2.4873(7) Å). In all three complexes, the central allyl carbon (meso-C) is closer to the metal center than are the terminal C atoms. For instance, in **1**·CH_2_Cl_2_, this distance is 2.210(3) Å and increases to 2.325(3) Å and 2.338(3) Å for the terminal C atoms. The average C–O bond lengths for the carbonyls in **1**·CH_2_Cl_2_ and **2** are very similar (∼1.155 Å) and are longer than that of 1.139(4) Å observed in **3**. Conversely, the corresponding M–C bonds are slightly shorter in **1**·CH_2_Cl_2_ and **2** (∼1.95 Å) than in **3** (1.984(3) Å), in line with the reduced π-back-donation to carbonyls in the last case, as supported by the analysis of the IR spectra. Notably, the bond angle between the equatorial N∩N donor atoms (N_1_–Mo–N_2_) remains nearly the same throughout the series. On the other hand, the bond angle defined by the equatorial CO ligands (C_1_–Mo–C_2/1′_) does change, being smallest (most compressed) for **2** (74.3(3)°), largest for **3** (80.64(14)°), and intermediate for **1**·CH_2_Cl_2_ (76.06(14)°).

DFT calculations,^34^ using the ADF program,^35−37^ were performed on the parent structures **1**–**3** and all their possible derivatives described below. They have revealed that the equatorial (type A) isomer is indeed always preferred over the axial (type B) isomer. The energy difference (in kcal mol^–1^) increases on going from **1** (5.23) to **2** (7.92), reflecting the extra stabilization of the equatorial isomer induced by the influence of the extra methyl group in 2-methallyl (Table S3 in the Supporting Information).

The energy difference between the two isomers of **3** is only 1.72 kcal mol^–1^. This is the first known complex of an *N*-aryl-Bian ligand that does not prefer the axial isomer and is also the first example of a 2-methallyl complex with a *N*-aryl-Bian-type ligand. This unusual arrangement reflects the large repulsion between the methyl substituents of pTol-Bian and the 2-methyl substituent of the allylic ligand, which overcomes the tendency to avoid cis orientation between the CO and *N*-donor atoms and the steric effects occurring in the equatorial isomers.

The structural parameters (Table S2 in the Supporting Information) are well reproduced by DFT calculations (Table S4 in the Supporting Information).

### Cyclic Voltammetry

**Figure 2:**
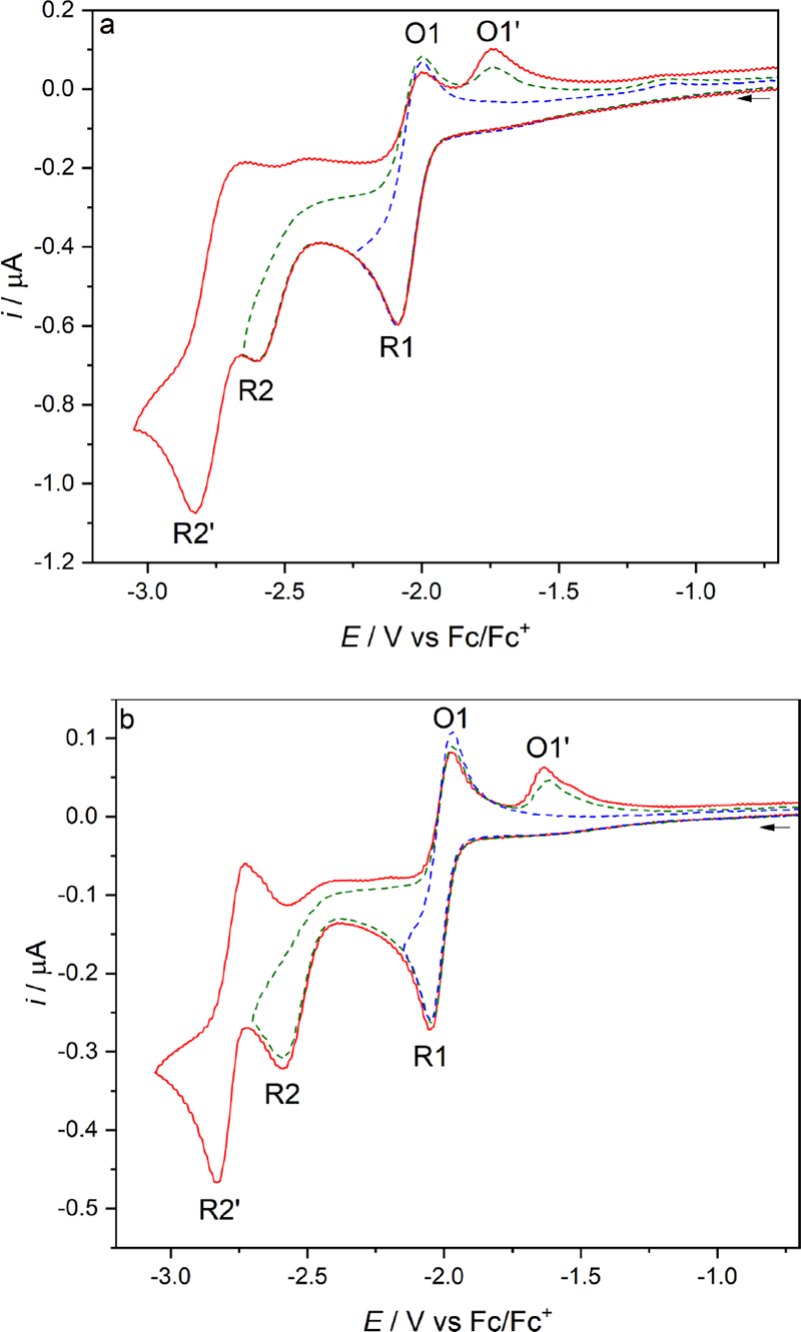
Cyclic voltammograms of complex **1** at (a) *T* = 298 K and (b) *T* = 195 K in THF/Bu_4_NPF_6_. The arrow indicates the initial scan direction. Conditions: Pt-microdisk electrode, υ = 100 mV s^–1^.

**Figure 3:**
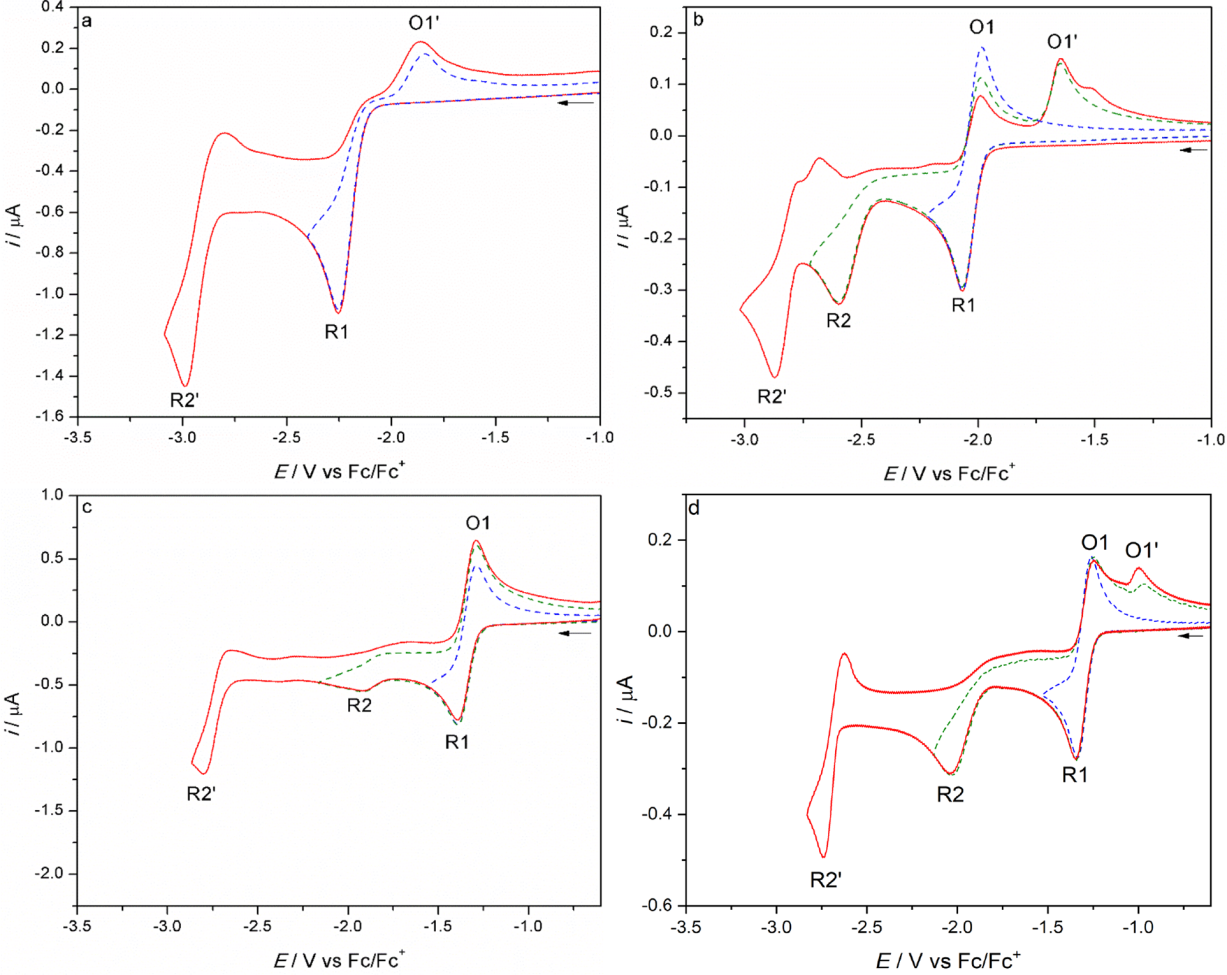
Cyclic voltammograms of complex **2** at (a) 298 K and (b) 195 K, and complex **3** at (c) 298 K and (d) 195 K in THF/Bu_4_NPF_6_. The arrow indicates the initial scan direction. Conditions: Pt-microdisk electrode, *v* = 100 mV s^–1^.

Cyclic voltammetry of **1**–**3** was conducted in argon-saturated THF/Bu_4_NPF_6_ (Figures 2 and 3 and Figure S12 in the Supporting Information) and PrCN/Bu_4_NPF_6_ (Figures S1–S3 and S13 in the Supporting Information) at 298 or 195 K on a Pt-microdisk electrode. The redox potentials determined for **1**–**3** are summarized in Table 1.

**Table 1:** Redox Potentials of Complexes **1**–**3** and Their Reduction Products (See Scheme 1) from Cyclic Voltammetry at a Pt-Microdisk Electrode at *T* = 298 K

	redox potential (V vs Fc/Fc^+^)				
solvent	Mo^II/III^ (*E*_1/2_)	R1 (*E*_1/2_)	R2 (*E*_p,c_)	R2′ (*E*_1/2_)	O1′ (*E*_p,a_)
[Mo(η^3^-allyl)(6,6′-dmbipy)(CO)_2_(NCS)]^a^					
THF	0.26	–2.02	–2.57	–2.94^b^	–1.84
THF^c^	0.28	–1.98	–2.60	–2.82	–1.66
PrCN	0.32	–1.93	–2.45	d	–1.73
PrCN^c^	0.38	–1.94	–2.56	d	–1.54
[Mo(η^3^-allyl)(6,6′-dmbipy)(CO)_2_Cl] (**1**)					
THF	0.16	–2.04	–2.61	–2.82^b^	–1.74
THF^c^	0.19	–2.01	–2.59	–2.78	–1.63
PrCN	0.16	–2.03	–2.60	–2.79^b^	–1.74
PrCN^c^	0.20	–1.99	–2.63	–2.83	–1.55
[Mo(η^3^-2-methallyl)(6,6′-dmbipy)(CO)_2_Cl] (**2**)					
THF	0.06	–2.25^b^	d	–2.98^b^	–1.83
THF^c^	0.10	–2.02	–2.60	–2.82	–1.64
PrCN	0.07	–2.14^b^	d	–2.89^b^	–1.71
PrCN^c^	0.10	–2.07	–2.66	–2.90	–1.61
[Mo(η^3^-2-methallyl)(pTol-Bian)(CO)_2_Cl] (**3**)					
THF	0.05	–1.34	–1.91	–2.80^b^	d
THF^c^	0.11	–1.29	–2.03	–2.64	–0.99
PrCN	0.04	–1.32	–1.93	–2.81^b^	–1.05
PrCN^c^	0.11	–1.28	–1.91	–2.72	–1.03

a Reference complex measured at an Au-microdisk electrode.^21^

b *E*_p,c_ value (anodic counter wave not observed).

c Measured at 195 K.

d Not observed.

At the CV level, the redox behavior of **1** closely resembles that already reported for [Mo(η^3^-allyl)(6,6′-dmbipy)(CO)_2_(NCS)].^21^ As the potential is swept positively, **1** undergoes a reversible, largely metal-based 1e^–^ oxidation to [Mo(η^3^-allyl)(6,6′-dmbipy)(CO)_2_Cl]^+^ at *E*_1/2_ = 0.16 V vs Fc/Fc^+^. The replacement of NCS^–^ with the stronger π-donor Cl^–^ lowers the oxidation potential by 100 mV in THF and 160 mV in PrCN.

In the negative potential region, there is a reversible 6,6′-dmbipy-based reduction (R1) at *E*_1/2_ = −2.04 V (THF) or −2.03 V (PrCN), producing the radical anion [Mo(η^3^-allyl)(6,6′-dmbipy)(CO)_2_Cl]^•–^ ([**1**]^•–^). As is the case for the NCS^–^ progenitor, there is no evidence for the formation of the 5-coordinate anion [Mo(η^3^-allyl)(6,6′-dmbipy)(CO)_2_]^−^ ([**1**-A]^−^) until the second, irreversible (R2) wave is passed at *E*_p,c_ = −2.61 V (THF) or −2.60 V (PrCN). The oxidation of [**1**-A]^−^ is then seen on the reverse anodic scan as a new anodic wave O1′ at *E*_p,a_ = −1.74 V. The final detectable cathodic process, R2′, at *E*_p,c_ = −2.82 V (THF) or −2.79 V (PrCN), corresponds to the partly reversible reduction of the 5-coordinate anion to the 5-coordinate dianion [**1**-A]^2–^. The cathodic behavior hardly changes at low temperature (195 K), although the R2′ wave becomes fully reversible and slightly shifts to *E*_1/2_ = −2.78 V (THF) or −2.83 V (PrCN).

In the positive potential region, **2** also undergoes a reversible metal-based oxidation to [**2**]^+^ at *E*_1/2_ = 0.06 V, which is less positively shifted than for **1** due to the stronger electron donation from the 2-methallyl group stabilizing **2**^+^. The cathodic behavior of **2** strongly differs from that of **1**, as the initial reduction in THF is a totally irreversible 2e^–^ (ECE) process occurring at *E*_p,c_ = −2.25 V (THF) or −2.14 V (PrCN). In comparison to **1** in THF, this corresponds to a ca. 150 mV negative shift of the parent reduction potential, as the replacement of allyl with 2-methallyl increases the LUMO energy. A similar negative potential shift was observed following the replacement of the bipy ligand with 4,4′-dmbipy.^21,49^

The anodic wave O1′, assigned to the oxidation of the 5-coordinate anion [**2**-A]^−^, is observed at *E*_p,a_ = −1.83 V (THF) or −1.71 V (PrCN) on the reverse scan that started directly beyond R1. There is only one other detectable cathodic wave, R2′, which is also shifted to the more negative potential of *E*_p,c_ = −2.98 V (THF) or −2.89 V (PrCN) and corresponds to the 1e^–^ reduction of [**2**-A]^−^ formed at R2. This behavior resembles that of [Mo(η^3^-allyl)(CO)_2_(4,4′-dmbipy)(NCS)];^21^ however, in this case there is no follow-up reduction of the dimer species [**2**-D] on the (sub)second CV time scale (i.e., no R(D) wave is detected). This means either that the dimer is reduced at the same electrode potential as for the parent complex (R1) (cf. [Mn(iPr-dab)(CO)_3_Br]^24^) or that the ultimate dimerization reaction (ECEC) is inhibited or too slow on the CV time scale to be observed. The first option is highly unlikely, given the large (almost 500 mV) separation between R1 and R(D) determined for the closely related complexes with the 4,4′ and 5,5′- dmbipy ligands.^21^ At *T* = 195 K, the initial R1 wave of **2** becomes fully reversible, with *E*_1/2_ = −2.02 V (THF) or −2.07 V (PrCN). The subsequent wave, R2, at *E*_p,c_ = −2.60 V (THF) and −2.66 V (PrCN), corresponds to the irreversible reduction of stable [**2**]^•–^, yielding the 5-coordinate anion [**2**-A]^−^. The latter reduces again to the corresponding 5-coordinate dianion at R2′ with *E*_1/2_ = −2.82 V (THF) and −2.90 V (PrCN).

Finally, complex **3** also undergoes a reversible metal-centered oxidation to [**3**]^+^ at *E*_1/2_ = 0.05 V vs Fc/Fc^+^, testifying to the donor power of the 2-methallyl and Cl^–^ ligands, capable of stabilizing the formal Mo(III) oxidation state, despite the significantly increased π-acceptor capacity of the pTol-Bian ligand in comparison to 6,6′-dmbipy. This anodic behavior is quite remarkable when it is compared to closely related reference systems, such as [Mo(η^3^-allyl)(2,6-xylyl-Bian)(CO)_2_(NCS)] that becomes irreversibly oxidized at ca. 0.6 V vs Fc/Fc^+^, a positive potential shift of more than 500 mV.^20^ Then, **3** is reversibly reduced to [**3**]^•–^ at much less negative potentials in comparison to **1** or **2**: viz., *E*_1/2_ = −1.34 V (THF) and −1.32 V (PrCN). This marked stabilization of the LUMO of **3** is fully consistent with the increased π-acceptor capacity of the pTol-Bian ligand. However, this reduction potential is still more negative than the value determined for the above Mo(2,6-xylyl-Bian)(NCS) reference that is already reversibly reduced at *E*_1/2_ = −1.16 V (THF). The radical anion [**3**]^•–^ is further reduced at R2, which lies at *E*_p,c_ = −1.91 V in THF (Figure 3c) and −1.93 V in PrCN (Figure S3 in the Supporting Information). The cathodic wave R2 is remarkably poorly resolved at room temperature in both solvents in comparison to the reduction of [**1**]^•–^ and [**2**]^•–^.

At *T* = 195 K, the CV response of **3** at negative potentials closely resembles the courses recorded for **1** and **2**. In comparison to the scans at room temperature, R1 shows a totally reversible shape comparable with that of the internal ferrocene standard. The irreversible wave R2 due to [**3**]^•–^ reduction shifts slightly negatively to −2.03 V in THF and becomes well developed in both THF and PrCN. This temperature-dependent behavior indicates some reorientation of [**3**]^•–^ at the cathodic surface at ambient temperature. This cathodic step generates the genuine 5-coordinate anion, [**3**-A]^−^, which is oxidized on the reverse anodic scan at O1′, *E*_p,a_ = −0.99 V (THF) or −1.03 V (PrCN), and reduced at R2′ to the corresponding stable dianion. The much larger separation between R2 and R2′ for **3** in comparison to **1** and **2** (Table 1) may reflect coordination of the donor solvent to [**3**-A]^−^, producing [**3**-Sv]^−^ (Sv = THF, PrCN), as revealed by IR spectroelectrochemistry and DFT calculations (see the following sections).

### Computational Studies

**Figure 4:**
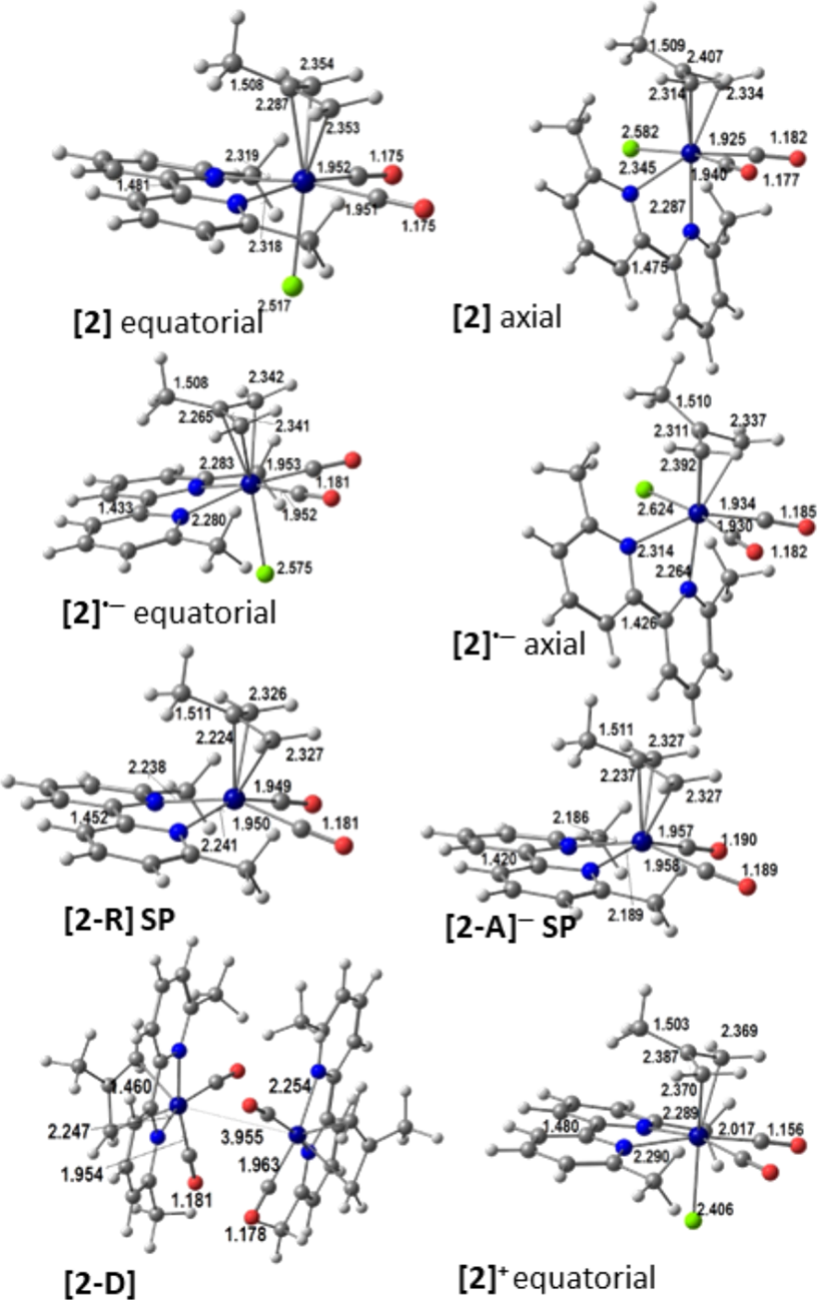
DFT-optimized structures of, from top to bottom, the parent complex [Mo(η^3^-6,6′-dmbipy)(CO)_2_Cl] (**2**) (the equatorial and axial isomers), the 1e^–^-reduced radical anion [**2**]^•–^ (the equatorial and axial isomers), the 5-coordinate radical [**2**-R] and 2e^–^-reduced 5-coordinate anion [**2**-A]^−^, the dimer [**2**-D], and the cation [**2**]^+^ with the relevant bond lengths (Å).

DFT calculations were performed to determine the ground-state geometries, electronic structures and energies, and to reproduce the vibrational spectra of complexes **1**–**3** and their oxidized and reduced forms introduced in the preceding CV section. The geometry-optimized structures are depicted in Figure 4 for **2** and in Figures S4 and S5 in the Supporting Information for **1** and **3**, respectively. The equatorial isomer is the most stable one for all of the neutral parent complexes, as discussed above.

The calculated IR ν(CO) wavenumbers are practically identical (Table S5 in the Supporting Information) for **1** and **2**, with the symmetric ν(CO) modes at 1878 and 1879 cm^–1^, respectively, and the antisymmetric modes at 1797 cm^–1^ in both cases. The experimental wavenumbers 1945 and 1861 cm^–1^ (in THF) for **1** are converted into 1886 and 1805 cm^–1^ by the application of a 0.97 scaling factor, in good agreement with the calculated values. For **3**, the calculated wavenumbers are somewhat larger, with the symmetric mode at 1891 cm^–1^ and the antisymmetric mode at 1821 cm^–1^. It is important to apply the scaling factor to calculated ν(CO) values for identification purposes of all studied 6-coordinate complexes (Table 2). It is redundant for the strongly π-delocalized 5-coordinate anions, [**X**-A]^−^.

**Table 2:** IR ν(CO) Absorption Data for Complexes **1**–**3** and Their Reduction Products (cf. Scheme 1)^a^

	ν(CO)/cm^–1^		ν(CN)/cm^–1^	
complex	exptl	DFT^n^	exptl	DFT
[Mo(η^3^-allyl)(6,6′-dmbipy)(CO)_2_(NCS)]^b^^–^d	1944, 1860		2082	
[Mo(η^3^-allyl)(6,6′-dmbipy)(CO)_2_(NCS)]^d^^,^^e^	1948, 1866	1881, 1800	2074	2054
[Mo(η^3^-allyl)(6,6′-dmbipy)(CO)_2_Cl] (**1**)^e^	1945, 1861	1878, 1797		
[Mo(η^3^-allyl)(6,6′-dmbipy)(CO)_2_Cl] (**1**)^b^^,^^c^	1940, 1854			
[Mo(η^3^-2-methallyl)(6,6′-dmbipy)(CO)_2_Cl] (**2**)^e^	1944, 1861	1879, 1797		
[Mo(η^3^-2-methallyl)(6,6′-dmbipy)(CO)_2_Cl] (**2**)^e^^,^^f^	1943, 1859			
[Mo(η^3^-2-methallyl)(6,6′-dmbipy)(CO)_2_Cl] (**2**)^b^,^c^	1940, 1853			
[Mo(η^3^-2-methallyl)(pTol-Bian)(CO)_2_Cl] (**3**)^e^	1956, 1886	1891, 1821		
[Mo(η^3^-2-methallyl)(pTol-Bian)(CO)_2_Cl] (**3**)^b^^,^^c^	1948, 1866			
[Mo(η^3^-allyl)(6,6′-dmbipy)(CO)_2_Cl]^+^ e	2053, 2000			
[Mo(η^3^-2-methallyl)(6,6′-dmbipy)(CO)_2_Cl]^+^ e	2053, 2000			
[Mo(η^3^-2-methallyl)(pTol-Bian)(CO)_2_Cl]^+^ e	2061, 2009			
[Mo(η^3^-allyl)(6,6′-dmbipy)(CO)_2_(NCS)]^•–^ b^–^d	1920, 1829	1855, 1764	2089	2069
[Mo(η^3^-allyl)(6,6′-dmbipy)(CO)_2_Cl]^•–^ b^,^^c^	1916, 1821	1852, 1759		
[Mo(η^3^-2-methallyl)(pTol-Bian)(CO)_2_Cl]^•–^ b^,^^c^	1928, 1836	1858, 1758		
[Mo(η^3^-allyl)(4,4′-dmbipy)(CO)_2_]_2_^d^		1858, 1844, 1787		
[Mo(η^3^-allyl)(6,6′-dmbipy)(CO)_2_]_2_^d^		1855, 1847, 1782		
[Mo(η^3^-2-methallyl)(6,6′-dmbipy)(CO)_2_]_2_		1855, 1847, 1782		
[Mo(bipy)(CO)_3_Y]^−^ g	1891, 1778, 1757			
[Mo(4,4′-dmbipy)(CO)_3_Y]^−^ d	1891, 1766, 1759			
[Mo(6,6′-dmbipy)(CO)_3_Y]^−^	1887, 1763, 1744			
[Mo(ptapzpy)(CO)_3_Br]^−^ h	1896, 1764, 1742			
[Mo(Xyl-dad)(CO)_3_Cl]^−^ i	1895, 1799, 1774			
[Mo(η^3^-allyl)(6,6′-dmbipy)(CO)_2_]^−^ b^,^^c^	1797, 1700^j^	1804, 1702^k^^,^^o^		
[Mo(η^3^-allyl)(6,6′-dmbipy)(CO)_2_]^−^ e	1795, 1720			
[Mo(η^3^-2-methallyl)(6,6′-dmbipy)(CO)_2_]^−^ b^,^^c^	1782, 1683^j^			
[Mo(η^3^-2-methallyl)(6,6′-dmbipy)(CO)_2_]^−^ e,f	1784, 1683	1802, 1701^o^		
[Mo(η^3^-2-methallyl)(6,6′-dmbipy)(CO)_2_]^−^ e	1789, 1710			
[Mo(η^3^-allyl)(4,4′-dmbipy)(CO)_2_(PrCN)]^−^ b^,^^d^	1896, 1797	1797, 1705		
[Mo(η^3^-2-methallyl)(pTol-Bian)(CO)_2_(PrCN)]^−^ b^,^^c^	1890, 1793	1832, 1738		
[Mo(η^3^-2-methallyl)(pTol-Bian)(CO)_2_(THF)]^−^ e	1897, 1800	1827, 1734		
[Mo(bipy)(CO)_3_]^2–^ g	1844, 1723, 1708			
[Mo(bipy)(CO)_3_]^2–^ l	1846, 1725, 1706			
[Mo(6,6′-dmbipy)(CO)_3_]^2–^	1843, 1708, 1694			
[Mo(6,6′-dmbipy)(CO)_3_]^2–^ m	1843, 1718, 1701			

a Key reference compounds are also included.

b Measured in PrCN.

c Measured at 223 K.

d Reproduced from ref (21).

e Measured in THF.

f Measured at 255 K.

g Reproduced from ref (20).

h Reproduced from ref (51).

i Reproduced from ref (52).

j Broad absorption bands.

k Derived from the equatorial isomer.

l Reproduced from ref (15).

m Reproduced from ref (23).

n Without the scaling factor (0.97).

o Scaling not needed for the strongly π-delocalized 5-coordinate anions.

**Figure 5:**
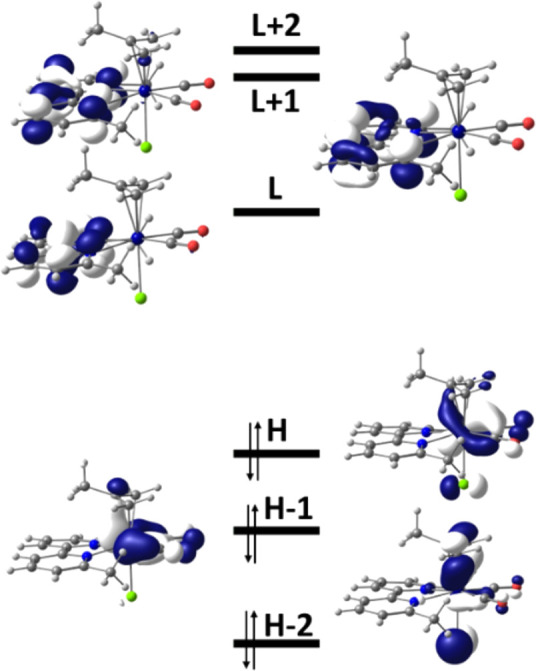
Frontier orbitals of the complex [Mo(η^3^-2-methallyl)(6,6′-dmbipy)(CO)_2_Cl] (**2**). Energies (eV): HOMO (H) −4.91, LUMO (L) −3.26.

As depicted in Figure 5 for **2** and Figures S6 and S7 in the Supporting Information for **1** and **3**, respectively, the HOMOs of **1**–**3** have a strong contribution from the metal, being bonding between the metal and the π-acceptor carbonyls, but π-antibonding between the Mo center and the π-donor chloride ligand. Hence, the 1e^–^ oxidation reaction, described in the preceding CV section, converts formally Mo(II) to Mo(III). The HOMO-1 and HOMO-2 do not differ significantly. Conversely, the LUMO, LUMO+1, and LUMO+2 are almost completely localized on the 6,6′-dmbipy ligand in **1** and **2** but only partially localized on the pTol-Bian ligand in **3**, indicating that the initial reduction step influences these ligands.

The localization of the initial reduction at the α-diimine ligand is reflected by the shortening of the 6,6′-dmbipy inter-ring bond, for example, from 1.481 to 1.433 Å in [**1**]^•–^ and from 1.481 to 1.442 Å in [**2**]^•–^ (see Table S4). On the other hand, the oxidation process does not affect this bond, which remains at 1.480 Å in both [**1**]^+^ and [**2**]^+^. The preference of the three complexes for the equatorial isomer is still observed in the singly reduced state, stabilized by 5.83 kcal mol^–1^ for [**1**]^•–^, 9.00 kcal mol^–1^ for [**2**]^•–^, and 2.39 kcal mol^–1^ for [**3**]^•–^. The calculated IR ν(CO) wavenumbers for the symmetric mode of the radical anions shift to the red by 26–33 cm^–1^, while the shifts for the antisymmetric mode are somewhat larger, ranging from 36 to 63 cm^–1^.

The loss of the chloride ligand from the primary 6-coordinate radical anion affords the 5-coordinate radical, [**X**-R], which in principle may adopt either a square-planar geometry (SP), derived from the equatorial isomer, or a trigonal-bipyramidal geometry (TBP), derived from the axial isomer.^21^ In these species, the SP geometry minimizes steric constraints between substituents and is preferred for [**1**-R], [**2**-R], and [**3**-R]. Only for [**1**-R] is a TBP geometry less stable by 1.56 kcal mol^–1^ also possible, suggesting that the 2-methyl group in the allyl plays a relevant role in the control of the metal coordination environment.

The direct reduction of the 5-coordinate radicals affords the active 2e^–^ catalysts for these systems, the 5-coordinate anions [**X**-A]^−^. The latter may, in principle, exist in either a closed-shell singlet (diamagnetic) or an open-shell triplet (paramagnetic) state. As in the previous cases,^21^ the former state is more stable by a high margin: 16.48 kcal mol^–1^ (**1**), 19.17 kcal mol^–1^ (**2**), and 11.77 kcal mol^–1^ (**3**). The three 5-coordinate anions adopt an SP geometry.

These SP 5-coordinate anions may react with coordinating solvents such as PrCN to form new 6-coordinate anionic complexes. The equatorial isomer forms easily from the SP precursor, by accepting the ligand electrons in the well oriented LUMO+1 (Figure S9 in the Supporting Information). However, this geometry is only found for [**1**-PrCN]^−^. Both [**2**-PrCN]^−^ and [**3**-PrCN]^−^ adopt the axial isomer geometry (it is shown in Figure S5 for the latter). These derivatives are not very stable, probably due to the negative charge in the acceptor fragment. In particular, the large reorganization of the Mo(η^3^-2-methallyl)(6,6′-dmbipy)(CO)_2_ fragment required to form [**2**-PrCN]^−^ makes the formation of this species very unlikely. The steric constraints imposed by the pTol-Bian and 2-methallyl ligands seem to be more important and the fragments barely reorganize when the sixth ligand (Cl or PrCN) adds, therefore allowing for the formation of the solvent complex.

**Figure 6:**
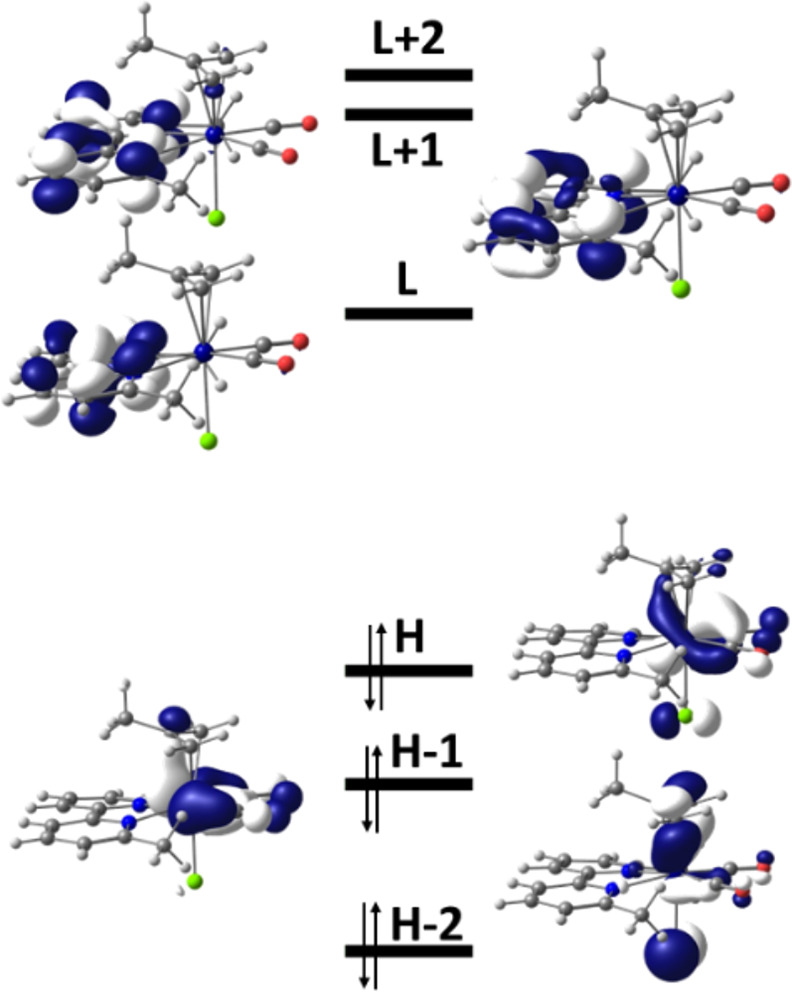
Frontier orbitals of the 5-coordinate anion [Mo(η^3^-2-methallyl)(6,6′-dmbipy)(CO)_2_]^−^ ([**2-**A]^−^). Energies (eV): HOMO (H) −2.86, LUMO (L) −1.61.

For the 6,6′-dmbipy complexes **1** and **2**, the loss of the chloride ligand from the radical anions, forming the 5-coordinate radicals, [**X**-R], has an almost negligible effect on the calculated IR ν(CO) wavenumbers. For instance, they are calculated to be 1851 and 1760 cm^–1^ for [**2**]^•–^ and 1857 and 1759 cm^–1^ for [**2**-R] (see Table S5 in the Supporting Information). The origin of this phenomenon has already been discussed.^21^ A more dramatic effect is calculated on proceeding from [**X**-R] to the 2e^–^-reduced 5-coordinate anions, [**1**-A]^−^ and [**2**-A]^−^, with a red shift of ν(CO) exceeding 55 cm^–1^. The lower symmetry of the 5-coordinate radicals and anions, in comparison to that of the parent 6-coordinate complexes and the corresponding radical anions, promotes mixing of orbitals, leading to electron delocalization, and noticeable changes in the bond lengths. For instance, in [**X**-R] the C–C′ inter-ring bonds lengthen slightly, while the Mo–C(allyl) bonds shorten. This effect is enhanced by the strong effect of the second electron added and can be observed in the frontier orbitals of [**2**-A]^−^ depicted in Figure 6 and [**1**-A]^−^ depicted in Figure S8 in the Supporting Information. In particular, the HOMO, LUMO, and LUMO+2 are strongly delocalized over the Mo-dmbipy unit, while LUMO+1 and HOMO-1, HOMO-2 are predominantly dmbipy and Mo(carbonyls) localized, respectively.

The bonding situation in 5-coordinate anion [**3**-A]^−^ is notably different, reflecting the different nature of its frontier orbitals in comparison to [**2**-A]^−^, as shown also in Figure S9 in the Supporting Information. The LUMO of [**3**-A]^−^ is almost exclusively (92%) localized on the pTol-Bian ligand, while the Mo center contributes only 19% to the HOMO. The latter value is significantly smaller than the contribution of Mo to the HOMO of [**2**-A]^−^ (29%). The calculations thus reveal that the added two electrons reside more on the pTol-Bian ligand than on the Mo center. This explains why the ν(CO)_s,a_ red shifts of ca. 41 and 47 cm^–1^ on going from [**3**-R] to [**3**-A]^−^ are smaller than those of 47/55 and 57/58 cm^–1^ calculated for the corresponding 6,6′-dmbipy complexes, [**1**-A]^−^ and [**2**-A]^−^ (Table S5 in the Supporting Information). The HOMO–LUMO electronic transition in 5-coordinate [**3**-A]^−^ exhibits an unusual ILET/MLCT character, remarkably different from the strongly delocalized π–π* (Mo-dmbipy based) character in [**2**-A]^−^. It is therefore possible that the more electron deficient Mo center in 16-VE [**3**-A]^−^ binds a donor solvent molecule. The resulting 6-coordinate [**3**-PrCN]^−^ is characterized by ν(CO) calculated wavenumbers of 1832 and 1738 cm^–1^. This behavior is revealed by the IR SEC experiments presented in the next section and previously reported^20^ for the 2e^–^ cathodic path of the closely related complex [Mo(η^3^-allyl)(2,6-xylyl-Bian)(CO)_2_(NCS)].

The two complexes of 6,6′-dmbipy, **1** and **2**, formed dimers with a long and weak Mo–Mo bond (3.886 Å in [**1**-D] and 3.955 Å in [**2**-D]), as shown in Figure 4 and Figure S4 in the Supporting Information. Their IR spectra are characterized by three strong ν(CO) bands appearing at 1855, 1847, and 1782 cm^–1^ for both Mo–Mo-bound dimers. No dimer of this type could be obtained from calculations for **3**.

### IR Spectroelectrochemistry at Low Temperature

IR spectroelectrochemistry has been proven to be an invaluable tool for unraveling the mechanistic details of different cathodic paths. The data presented in this section support the major insights gained from the cyclic voltammograms and DFT calculations in the preceding sections. The IR ν(CO) absorption data recorded for parent **1**–**3**, their oxidized and reduced products, and key reference compounds are summarized in Table 2 (and complemented with relevant DFT data taken from Table S5 in the Supporting Information). It is convenient to begin the discussion with the cathodic paths of **1**–**3**, determined at low temperature (223 K), as these results are the most straightforward to assign.

**Figure 7:**
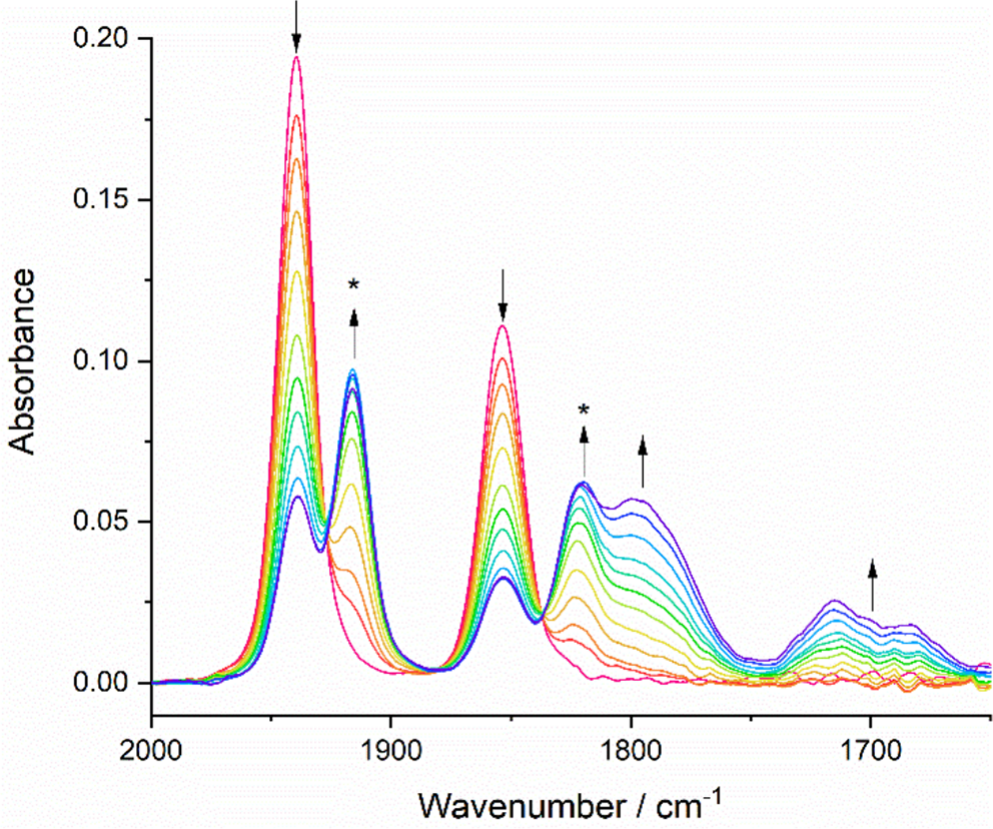
IR SEC monitoring of the reduction of [Mo(η^3^-allyl)(6,6′-dmbipy)(CO)_2_Cl] (**1**) (↓) at R1 to yield [**1**]^•–^ (*) and 5-coordinate [**1**-A]^−^ as the ultimate secondary product (↑). Conditions: a cryostated OTTLE cell, PrCN/Bu_4_NPF_6_, *T* = 223 K.

Reducing complex **1** (ν(CO): 1940, 1854 cm^–1^) at a potential coinciding with R1 in PrCN at 223 K (Figure 2b) converts the parent complex to a mixture of two products absorbing in the ν(CO) region (Figure 7). On the basis of a comparison with the complexes in ref (21), the two species are assigned as the primary radical anion [**1**]^•–^ (ν(CO): 1916, 1821 cm^–1^), accompanied (with some delay) by the 2e^–^ reduced 5-coordinate anion (the ECE path) [**1**-A]^−^ (ν(CO): 1797, 1700 cm^–1^). The large ν(CO) wavenumber shift when **1** is converted to [**1**-A]^−^ is consistent with the characteristic electron-rich, π-delocalized M−α-diimine structures of many related 5-coordinate anions, such as [Ru(Me)(CO)_2_(iPr-dab)]^−^ (ν(CO): 1913, 1832 cm^–1^) obtained by 2e^–^ reduction of [Ru(Me)(CO)_2_(iPr-dab)(I)] (ν(CO): 2027, 1960 cm^–1^); iPr-dab stands for *N,N′*-diisopropyl-1,4-diazabuta-1,3-diene.^50^ The calculated ν(CO) frequencies for [**1**]^•–^ (1909, 1813 cm^–1^, after dividing by 0.97) and [**1**-A]^−^ (1804, 1702 cm^–1^) reproduce well the experimental frequencies. In contrast to the ref (21) complex [Mo(η^3^-allyl)(6,6′-dmbipy)(CO)_2_(NCS)]^•–^, radical anion [**1**]^•–^ is unstable on the SEC time scale even at low temperature, despite the fully reversible cathodic wave R1 in the cyclic voltammogram (Figure 2). This is a consequence of the strong π-donation from the Cl^–^ ligand, which is less tunable than that of the isothiocyanate ligand via Mo^–^=N=C=S ↔ Mo–N≡C–S^–^.

**Figure 8:**
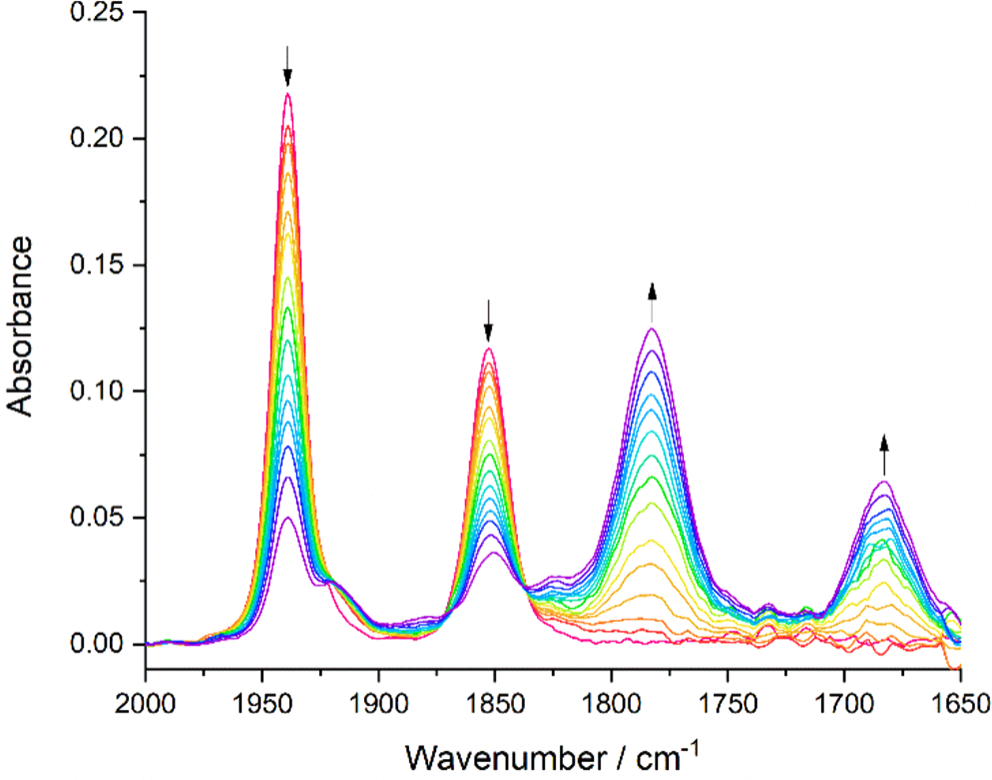
IR SEC monitoring of the overall 2e^–^ reduction of [Mo(η^3^-2-methallyl)(6,6′- dmbipy)(CO)_2_Cl] (**2**) (↓) at R1 to the 2e^–^ reduced 5-coordinate anion [**2**-A]^−^ (↑). Conditions: a cryostated OTTLE cell, PrCN/Bu_4_NPF_6_, *T* = 223 K.

In contrast to **1**, reducing 2-methallyl complex **2** in PrCN at the cathodic wave R1 under the same low-temperature conditions (Figure 8) results in its conversion to just a single species, [Mo(η^3^-2-methallyl)(6,6′-dmbipy)(CO)_2_]^−^ ([**2**-A]^−^), with smaller ν(CO) wavenumbers (1782, 1683 cm^–1^; calculated at 1802, 1701 cm^–1^) in comparison to [**1**-A]^−^. This red ν(CO) shift reflects the increased electron density at the CO ligands imposed by the 2-methallyl ligand, which has a stronger effect in [**2**-A]^−^ than in parent **2** due to the widely delocalized nature of the π-bonding in the 5-coordinate anion (see the preceding DFT section). These IR SEC results are consistent with the different CV behaviors of **1** and **2** (Figures 2 and 3, respectively), clearly confirming that the methylated allyl group significantly destabilizes the 1e^–^-reduced intermediate [**2**]^•–^. At low temperature, this results in the rapid formation of stable [**2**-A]^−^ already at R1 via [**2**-R] (Scheme 1); the dimerization is inhibited. At ambient temperature, however, the cathodic course in the thin-layer cell becomes more complex, as described in the next section.

**Figure 9:**
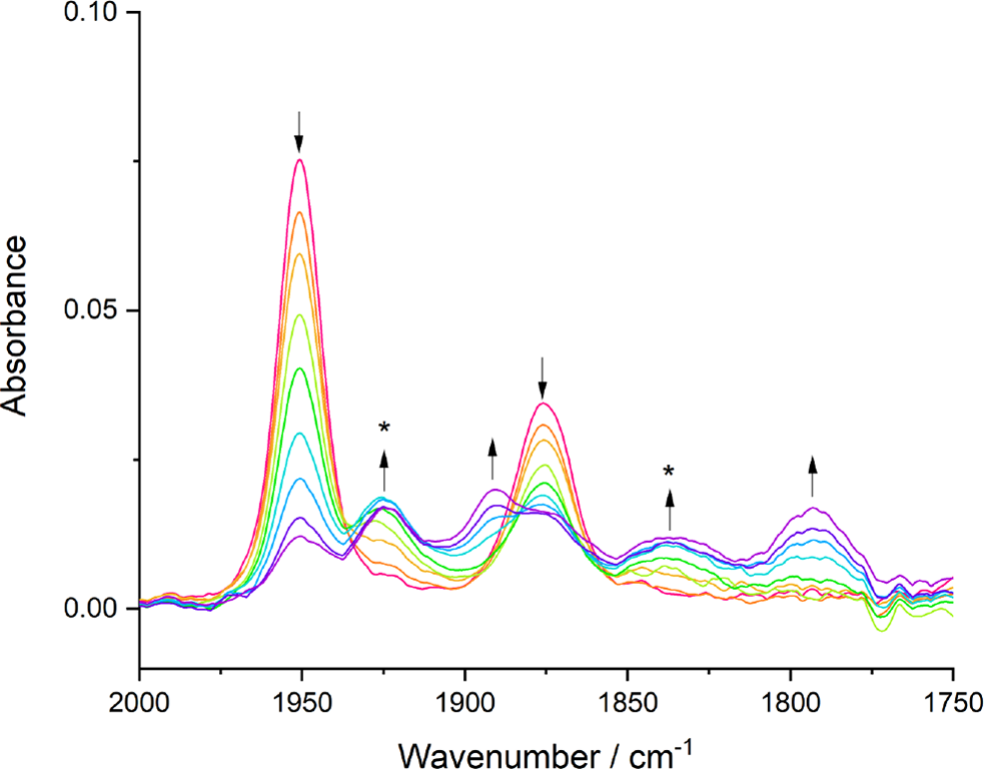
IR SEC monitoring of the reduction of 2 mM [Mo(η^3^-2-methallyl)(pTol-Bian)(CO)_2_Cl] (**3**) (↓) at R1, resulting in a mixture of [**3**]^•–^ (*) and 2e^–^ reduced 6-coordinate anion [**3**-PrCN]^−^ (↑). Conditions: a cryostated OTTLE cell, PrCN/Bu_4_NPF_6_, *T* = 223 K.

Perhaps most surprising in the studied series is the low-temperature cathodic behavior of **3** (ν(CO): 1951, 1876 cm^–1^). On the basis of the recorded CV responses and the strongly π-accepting nature of the *N*-aryl-Bian ligand, one would expect the corresponding radical anion, [**3**]^•–^, to persist in the electrolyte. However, the initial reduction of **3** at R1 generated a mixture of two species absorbing in the ν(CO) region (Figure 9), akin to the case for **1**. On comparison with the reference complexes (Table 2), they have been assigned as the minor radical anion (ν(CO): 1925, 1836 cm^–1^, calculated as 1915, 1812 cm^–1^ after dividing by 0.97) and the 6-coordinate solvento anion, [**3**-PrCN]^−^ (ν(CO): 1890, 1793 cm^–1^ calculated as 1889, 1792 cm^–1^ after dividing by 0.97), as a secondary product. This behavior is ascribed to the cooperative destabilizing donor effects of the Cl^–^ and 2-methallyl ligands. The ref (20) complex, [Mo(η^3^-allyl)(2,6-xylyl-Bian)(CO)_2_(NCS)], reduces to the stable radical anion already at room temperature. The cyclic voltammetric study of **3** in THF indicates that the reduction of [**3**]^•–^ at R2 generates the 5-coordinate anion [**3**-A]^−^ (Figure 3d), and the CV responses of **3** in PrCN do not show any substantial difference from this behavior (Figure S3 in the Supporting Information). Obviously, the strong coordinating ability of the PrCN solvent needs to be considered. The solvento anion [**3**-PrCN]^−^ is formed already at R1 (Figure 9), most likely from an equilibrium between [**3**]^•–^ and [**3**-R] reducible to [**3**-A]^−^ that coordinates a donor solvent molecule. Alternatively, [**3**-R] coordinates PrCN prior to the ultimate reduction. Such a cathodic behavior has been well documented: for example, for [Re(bipy)(CO)_3_Cl] in PrCN.^4^

### IR Spectroelectrochemistry at Ambient Temperature

**Figure 10:**
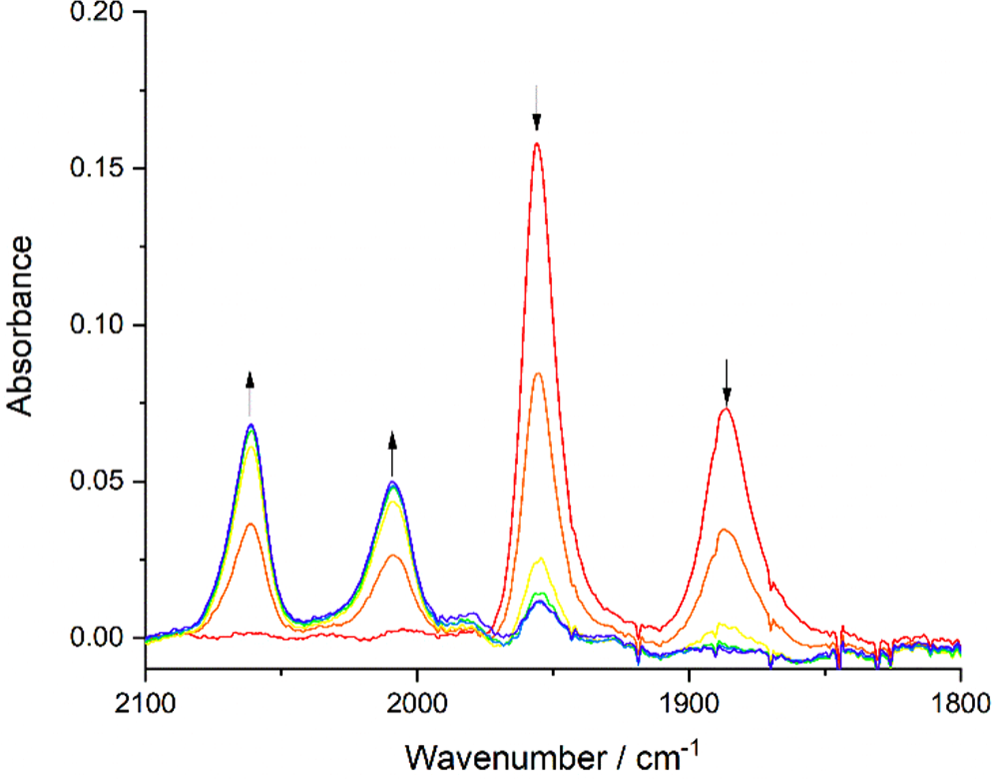
IR SEC monitoring of the 1e^–^ oxidation of [Mo(η^3^-2-methallyl)(pTol-Bian)(CO)_2_Cl] (**3**) (↓) to stable [**3**]^+^ (↑). Conditions: an OTTLE cell, THF/Bu_4_NPF_6_, *T* = 298 K.

In line with the ordinary reversible anodic cyclic voltammetric scans, both studied Mo–2-methallyl complexes **2** (Figure S10b, Supporting Information) and **3** (Figure 10) are oxidized on the SEC time scale to the corresponding stable, formally Mo(III) cationic products. On the other hand, [**1**]^+^ is unstable at room temperature (Figure S10a in the Supporting Information) and slowly decomposes (decarbonylates) during the electrolysis. The accompanying blue shifts of the ν(CO) bands (summarized in Table 2 and reflected in the DFT-calculated values) to larger wavenumbers are significant. They comply with the depopulation of the largely Cl–Mo-based HOMO of the parent complexes (Figure 5 and Figures S6 and S7 in the Supporting Information), having the expected large effect on the degree of CO π-back-donation that decreases in the formally Mo(III) products. The reversible oxidation of complex **3** is truly remarkable. The Mo–bipy bond lengths barely change upon oxidation, while the internal bonds in pTol-Bian display larger changes. Therefore, the stability of [**3**]^+^ is preserved. The same patterns are different for [**1**]^+^ and [**2**]^+^, where the dominant donor effect of the 2-methallyl ligand decides about stability of the latter cation.

**Figure 11:**
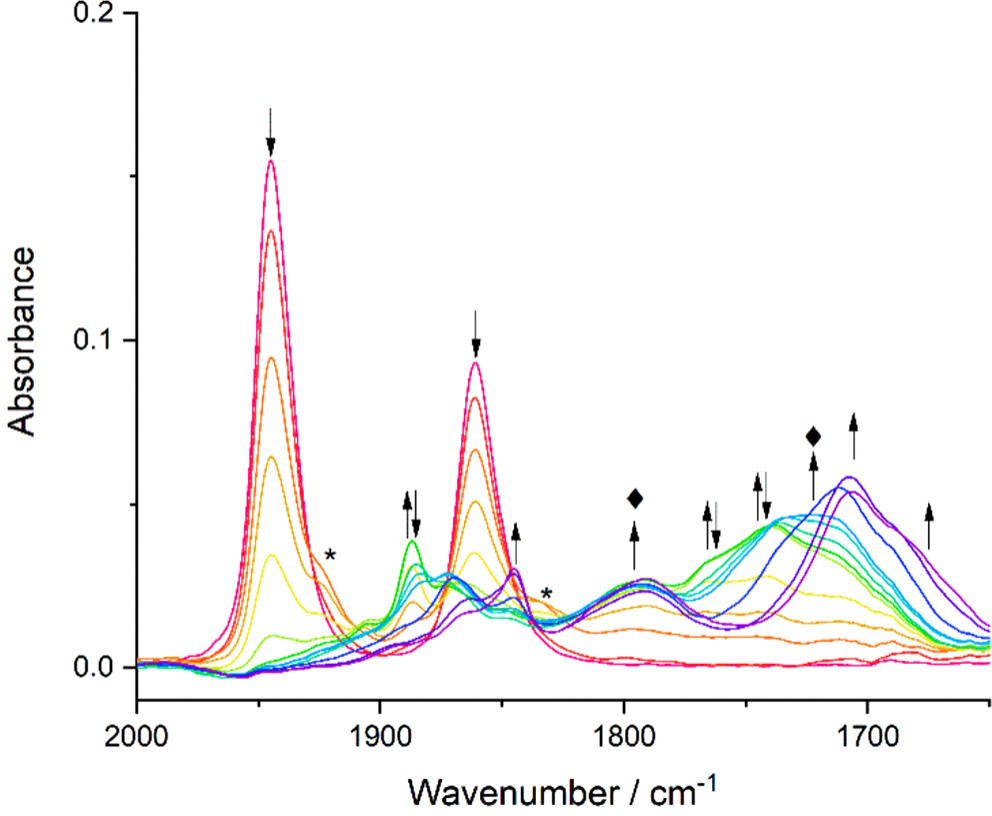
IR SEC monitoring of the reduction of [Mo(η^3^-allyl)(6,6′-dmbipy)(CO)_2_Cl] (**1**) (↓) at R1 generating a mixture of [Mo(η^3^-allyl)(6,6′-dmbipy)(CO)_2_]^−^ ([**1**-A]^−^) (⧫) and [Mo(6,6′-dmbipy)(CO)_3_Y]^−^ (↑↓). The subsequent reduction of the latter complex to [Mo(6,6′-dmbipy)(CO)_3_]^2–^ (↑) is also shown. The asterisk (*) indicates the minor intermediate absorption of [**1**]^•–^ as the primary reduction product (Figure 7). Conditions: an OTTLE cell, THF/Bu_4_NPF_6_, *T* = 298 K.

Conducting IR SEC in the negative potential region at ambient temperature in THF/Bu_4_NPF_6_ reveals additional complexity in the cathodic paths of **1** and **2** in comparison to the straightforward cathodic behavior seen at 223 K (see the preceding section). The reduction of **1** at R1 (Figure 11) leads to a mixture of products, a very minor component of which is the radical anion [**1**]^•–^. Initially, the mixture contains two major secondary products that can be identified from their ν(CO) stretching wavenumbers.

The initial secondary reduced compound (ν(CO): 1795, 1720 cm^–1^) detectable at ambient temperature can be assigned as the 2e^–^-reduced 5-coordinate anion [**1**-A]^−^ (Figure 11). In contrast to its exclusive formation upon cooling (Figure 7), [**1**-A]^−^ was accompanied by another species (ν(CO): 1887, 1760, 1738 cm^–1^), which is known^21^ to replace the reactive genuine dimer [Mo(η^3^-allyl)(6,6′-dmbipy)(CO)_2_]_2_ ([**1**-D]) (Scheme 1). The ν(CO) modes calculated with DFT for model [**1**-D] (Figure S4 in the Supporting Information) exhibit similar wavenumbers (1855, 1847, and 1782 cm^–1^) but differ from the ultimate secondary product in the three-band pattern: viz., 2 + 1 vs 1 + 2, respectively. The latter ν(CO) band pattern corresponds to a 6-coordinate facial tricarbonyl complex. In the literature, very similar ν(CO) wavenumbers (1896, 1764, 1742 cm^–1^) have been reported for the complex [Mo(ptapzpy)(CO)_3_Br]^−^ (ptapzpy = 2-(1-propyltrimethylammonium-3-pyrazolyl)pyridine).^51^ The closely related complex [Mo(Xyl-dab)(CO)_3_Cl]^−^ (Xyl-dab = *N,N′*-2,6-dimethylphenyl-1,4-diazabuta-1,3-diene) shows larger ν(CO) wavenumbers due to the less donating α-diimine ligand (Table 2).^52^ We denote the secondary product accompanying [**1**-A]^−^ as [Mo(6,6′-dmbipy)(CO)_3_Y]^−^ (herewith replacing the label [**1**-D′] adopted in the preceding paper^21^). The exact molecular structure of [Mo(6,6′-dmbipy)(CO)_3_Y]^−^ and the mechanism of its formation still remain to be resolved, presenting a challenge for preparative electrochemistry.^53^ The anionic ligand Y in [Mo(6,6′-dmbipy)(CO)_3_Y]^−^ can be the σ-bound allyl or the chloride released from reduced **1** in the initial cathodic step. The ECEC mechanism converting parent **X** via [**X**-A]^−^ to dimer [**X**-D] (Scheme 1) has been presented in detail in the previous study.^21^ The IR spectroelectrochemical detection of [Mo(6,6′-dmbipy)(CO)_3_Y]^−^ (Figure 11) proves indirectly the formation of [**1**-D] along the cathodic path of **1** at ambient temperature, regardless of the lack of evidence from cyclic voltammetry for the zero-electron reaction between [**1**-A]^−^ and **1** (see above). The subsequent reduction of [Mo(6,6′-dmbipy)(CO)_3_Y]^−^ does not regenerate [**1**-A]^−^. Instead, the ultimate reduction product is 5-coordinate [Mo(6,6′-dmbipy)(CO)_3_]^2–^ (ν(CO): 1843, 1708, 1694 cm^–1^; Table 2), which is the active catalyst in the photoassisted reduction of CO_2_ to CO.^23^

**Figure 12:**
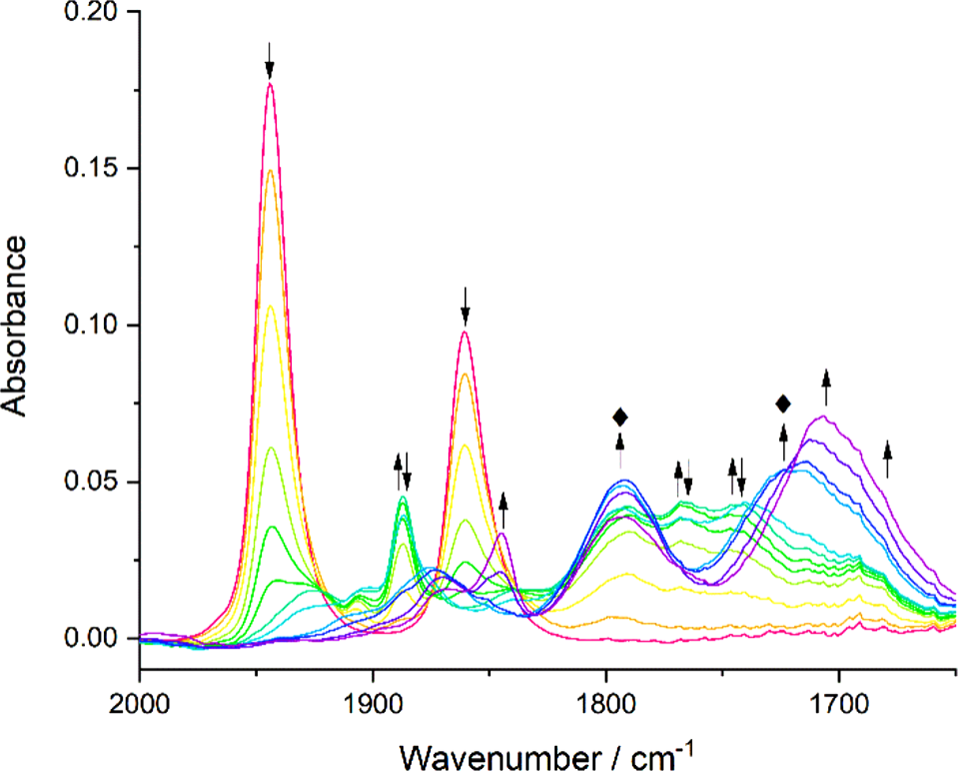
IR SEC monitoring of the reduction of [Mo(η^3^-2-methallyl)(6,6′-dmbipy)(CO)_2_Cl] (**2**) (↓) at R1 to the 5-coordinate anion, [**2**-A]^−^ (⧫) and [Mo(6,6′-dmbipy)(CO)_3_Y]^−^ (↑↓). The subsequent reduction of the latter complex to [Mo(6,6′-dmbipy)(CO)_3_]^2–^ (↑) is also shown. Conditions: an OTTLE cell, THF/Bu_4_NPF_6_, *T* = 298 K.

Electrochemical reduction of **2** (Figure 12) in THF at ambient temperature proceeds in a fashion very similar to that described above for **1**. The cathodic step R1 (Table 1) is irreversible, again leading to a mixture of 5-coordinate [**2**-A]^−^ and [Mo(6,6′-dmbipy)(CO)_3_Y]^−^ that is further reducible to [Mo(6,6′-dmbipy)(CO)_3_]^2–^. Importantly, the ν(CO) absorption bands belonging to the radical anion [**2**]^•–^ do not appear, confirming the increased reactivity of the primary reduction product at ambient temperature, in line with the irreversible CV cathodic wave R1 (Figure 3a). The 5-coordinate anion [**2**-A]^−^ (ν(CO): 1789, 1710 cm^–1^) exhibits slightly larger CO stretching wavenumbers in comparison to measured for free [**2**-A]^−^ in chilled THF (255 K, Figure S11 in the Supporting Information) and PrCN (223 K) electrolytes (Table 2), indicating that a weak adduct^21^ formed in the reaction mixture.

**Figure 13:**
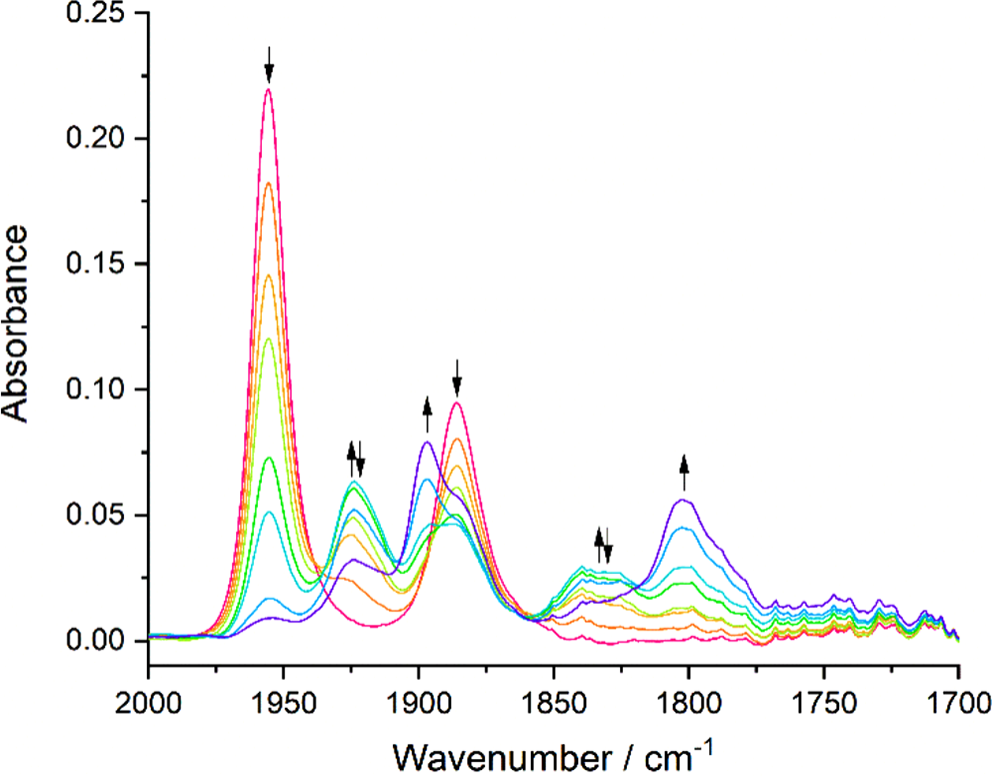
IR SEC monitoring of the reduction of [Mo(η^3^-2-methallyl)(pTol-Bian)(CO)_2_Cl] (**3**) (↓) at R1 to [**3**]^•–^ (↑↓) and 2e^–^-reduced 6-coordinate anion [**3**-THF]^−^ (↑) in a redox equilibrium. Conditions: an OTTLE cell, THF/Bu_4_NPF_6_, *T* = 298 K.

Electrochemical reduction of **3** in THF at ambient temperature (Figure 13) exhibits a cathodic behavior similar to that encountered for this complex in PrCN at low temperature. The initial reduction at R1 produces once more the unstable radical anion [**3**]^•–^, transforming to the solvated 6-coordinate anion [**3**-THF]^−^ (ν(CO): 1897, 1800 cm^–1^). Dimer [**3**-D] was neither observed on the SEC time scale nor could be calculated using approaches that led to dimers [**X**-D] for **1** and **2**. This is likely a result of the steric hindrance from the bulky pTol-Bian ligand destabilizing the dimer conformation.

The transient appearance of dimer [**1**-D] along the cathodic path of **1** might be considered highly surprising, as results (CV, SEC, DFT) from the previous series [Mo(η^3^-allyl)(*x,x′*-dmbipy)(CO)_2_(NCS)] (*x,x′* = 4–6) indicated that the most sterically demanding 6,6′-dmbipy ligand stabilized the 5-coordinate anion, [**1**-A]^−^, toward dimerization. This study, however, reveals that the true story is more complicated. The dimer is expected to form in a zero-electron coupling reaction between the 5-coordinate anion and the yet nonreduced parent complex (Scheme 1). Thus, the proclivity of the dimer formation is dependent on several factors. The first is the inertness of the parent complex itself. If the Mo–X (X = Cl, NCS) bond in the parent complex is weaker, then it is obviously more susceptible to this form of nucleophilic attack on the appropriate time scale, and the dimer therefore has a higher chance to form. We conclude firmly that the Mo–Cl bonds in **1** and **2** are weaker than the Mo–N(CS) bond in the reference complex [Mo(η^3^-allyl)(6,6′-dmbipy)(CO)_2_(NCS)]. Second, the stability of the 1e^–^-reduced intermediate, i.e. the radical anions [**1**]^•–^ and [**2**]^•–^, also plays a role. The more reactive Mo–Cl bond facilitates a greater amount of the 5-coordinate anions being available to react with the parent during the initial cathodic step, driving the reduction mechanism more along the pathway involving the dimer. This conclusion underlines the need to determine the exact mechanism of the rapid concomitant conversion of [**1**-D] to [Mo(6,6′-dmbipy)(CO)_3_Y]^−^ on the time scale of IR spectroelectrochemistry. The cathodic pathways described in this study have a strong effect on the results gathered during electrochemical reduction under a CO_2_ atmosphere, which are presented in the next section.

### Cyclic Voltammetry and IR Spectroelectrochemistry under a CO_2_ Atmosphere

**Figure 14:**
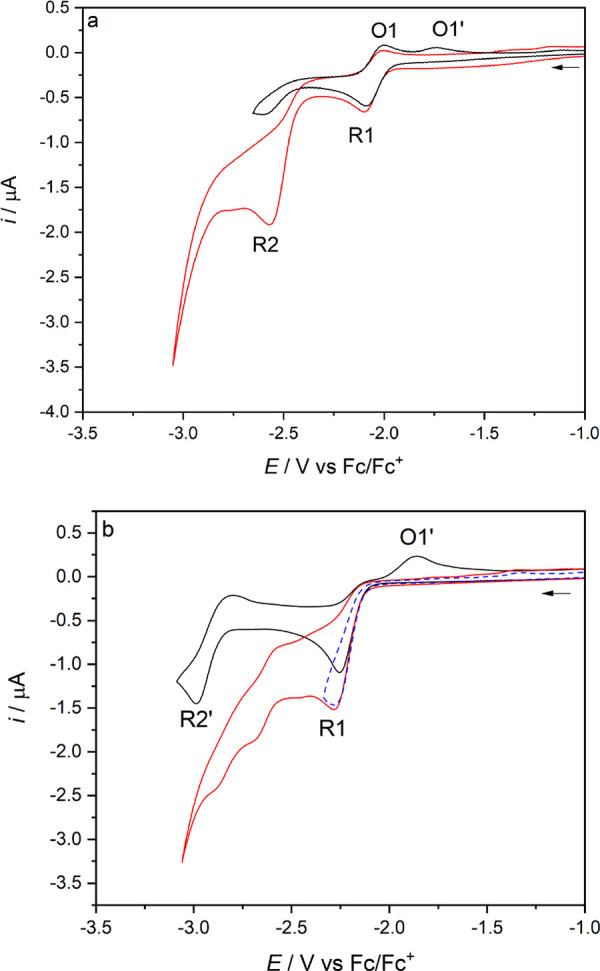
Cyclic voltammograms of complexes **1** (a) and **2** (b) in THF/Bu_4_NPF_6_ saturated with CO_2_ (red and dashed blue curves) and argon (reference black curves). Conditions: Pt-microdisk electrode, *v* = 100 mV s^–1^, *T* = 298 K.

The CV studies of **1** and **2** in THF were repeated under an atmosphere of CO_2_ (Figure 14) in order to probe for any catalytic activity of 5-coordinate anions [**X**-A]^−^ (**X** = **1**, **2**) along the cathodic paths toward the 2e^–^ catalytic reduction of CO_2_. For complex **1**, the 1e^–^ cathodic wave R1 (cf. Figure 2) remains unchanged, producing stable [**1**]^•–^. However, catalytic current enhancement is observed at the R2 wave, where [**1**-A]^−^ is produced via the subsequent reduction of the radical anion. On the reverse anodic scan, the wave O1′, which corresponds to [**1**-A]^−^ reoxidation, is absent, confirming the rapid interaction of the 5-coordinate anion with CO_2_. It should be recalled, however, that [**1**-A]^−^ forms already at R1 on the IR spectroelectrochemical time scale (Figure 7).

For the CV of complex **2**, the behavior is different. Interestingly, a modest increase in the cathodic current is observed already at R1, which most likely corresponds to the catalytic reduction of CO_2_ by [**2**-A]^−^ that has already been identified as the dominant product at this wave on the CV time scale (Figure 3a). Correspondingly, the anodic counter wave O1′ is absent on the reverse anodic scan starting directly beyond R1. However, the bulk of the catalytic current enhancement is not seen until slightly more negative potentials are reached, where also a new quasi-reversible wave is detected at ca. −2.7 V. The latter may correspond to reduction of an unreactive intermediate adduct of [**2**-A]^−^ and CO_2_. For example, an unreactive bicarbonate complex was encountered for [Mn(mesityl-bipy)(CO)_3_]^−^ and [Mn(iPr-dab)(CO)_3_]^−^ catalysts under a CO_2_ atmosphere.^25,54^

Under the same conditions, **3** was not catalytically active toward the CO_2_ substrate along the cathodic CV scan, which is consistent with previous observations on the poor catalytic performance of a closely related Mo–allyl complex with 2,6-dimethylphenyl-Bian.^20^ Indeed, IR spectroelectrochemistry in the preceding section has provided no evidence for the cathodic generation of 5-coordinate [**3**-A]^−^ undergoing an electrophilic attack by CO_2_.

**Figure 15:**
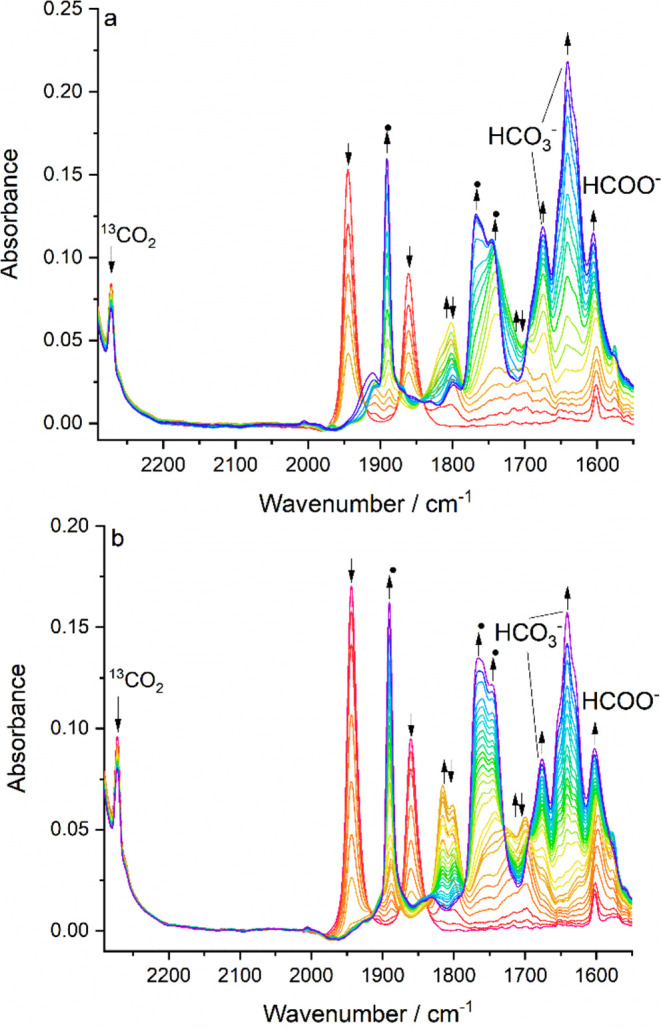
IR spectral responses of **1** (a) and **2** (b) to their reduction in CO_2_-saturated THF/Bu_4_NPF_6_, showing the conversion of the parent complex (↓) to the adduct [**X**···CO_2_]^−^ (**X** = **1**,**2**) (↑↓) and the concomitant cathodic process resulting in CO/bicarbonate and formate, as well as inactive [Mo(6,6′-dmbipy)(CO)_3_Y]^−^ (**●**). Conditions: Pt-minigrid electrode, an OTTLE cell, *T* = 298 K.

IR spectroelectrochemistry was conducted with **1** and **2** to monitor the reduction path in CO_2_-saturated THF/Bu_4_NPF_6_ (Figure 15). These long-lasting spectroelectrochemical experiments reveal hardly any difference between the electrocatalytic abilities of **1** and **2**. For both complexes, the initial reduction at R1 does not generate 5-coordinate [**X**-A]^−^ but its weak adducts with CO_2_ formulated^21^ as [**X**···CO_2_]^−^ (ν(CO): 1810, 1720 cm^–1^ and 1795, 1698 cm^–1^) and a lesser amount of inactive [Mo(6,6′-dmbipy)(CO)_3_Y]^−^ (ν(CO): 1891, 1764, 1746 cm^–1^). DFT calculations led to identification of the stable 6-coordinate [**X**-CO_2_]^−^ with larger ν(CO) stretching wavenumbers: 1829, 1741 cm^–1^ (**X** = **1**) and 1830, 1742 cm^–1^ (**X** = **2**; Figure 4). This strong 6-coordinate adduct with CO_2_ was observed experimentally for the 4,4′-dmbipy ligand.^21^ As the reduction potential is swept more negatively, both [**X**···CO_2_]^−^ adducts convert further to [Mo(6,6′-dmbipy)(CO)_3_Y]^−^, which represents a deactivation route for these catalysts. The catalytic conversion of CO_2_ within the OTTLE cell during the cathodic scan is moderate, as revealed by the decreasing reference ^13^CO_2_ peak at 2275 cm^–1^. The products are in both cases free formate absorbing at 1607 cm^–1^ ^55^ accompanied by bicarbonate (1674 and 1649 cm^–1^) and free CO in an amount not detectable in the IR spectra. However, the formation of an excess of CO explains why the tricarbonyl complex [Mo(6,6′-dmbipy)(CO)_3_Y]^−^ forms almost quantitatively when the electrochemical reduction of both **1** and **2** is conducted under a CO_2_ atmosphere.

## Conclusions

This work strongly supports our ongoing efforts to characterize the fascinating redox reactivity of the formally Mo(II) complexes [Mo(η^3^-2-R-allyl)(α-diimine)(CO)_2_X] (X = halide, pseudohalide). This study based on [Mo(η^3^-2-R-allyl)(6,6′-dmbipy)(CO)_2_Cl] (R = H, CH_3_) has resulted in several important discoveries. First, the interplay of steric and electronic effects between the various ligands (X = halide, pseudohalide; α-diimine; R-allyl) is more complex than was originally anticipated; it is also important to consider the effects of different time scales, in order to fully appreciate the whole situation. For instance, the replacement of the NCS^–^ ligand with Cl^–^ initially (when analyzing the CV scans) does not seem to affect the cathodic path strongly. On the other hand, IR SEC has revealed that there is actually a strong effect on the stability of the primary radical anions at ambient temperature and the reactivity of the ECE-generated, 2e^–^-reduced 5-coordinate anions toward the parent complexes, resulting in Mo–Mo dimerization. In contrast to the dimethyl-bipy substitution in the 6,6′-position, the substitution at the meso-carbon of the allyl ligand results in a strongly decreased stability of the radical anions toward the cleavage of the Mo–Cl bond. The new Cl^–^ and 2-methallyl ligand assembly studied in this work also eliminates the usually stabilizing influence of the π-acceptor pTol-Bian ligand on the singly reduced species, resulting not only in a different parent molecular structure (A-type) in comparison to other Mo–*N*-aryl-Bian complexes but also in the increased reactivity of the radical anion (even at low temperature).

The catalytic activity of the 2e^–^-reduced 5-coordinate anions [Mo(η^3^-2-R-allyl)(6,6′-dmbipy)(CO)_2_]^−^ toward the conversion of CO_2_ to CO and formate has been proven by CV and IR SEC. Both anions remain stable ultimate reduction products under argon only in chilled electrolyte solutions. At ambient temperature they attack the yet nonreduced parent complexes, forming reactive [Mo(η^3^-2-R-allyl)(6,6′-dmbipy)(CO)_2_]_2_. The dimerization step is relatively slow due to the sterically demanding 6,6′-dmbipy ligand (in comparison to 4,4′-dmbipy) and does not occur on the CV time scale. This enables the catalytic activity of [Mo(η^3^-2-methallyl)(6,6′-dmbipy)(CO)_2_]^−^ to be distinguished already at the parent cathodic wave R1 while the catalyst [Mo(η^3^-allyl)(6,6′-dmbipy)(CO)_2_]^−^ is generated at R2. On the longer time scale of IR SEC, both anions are generated already at R1. Their partial conversion to [Mo(η^3^-2-R-allyl)(6,6′-dmbipy)(CO)_2_]_2_ (modeled by DFT), generally corresponding to the ECEC route evidenced by cyclic voltammetry (for 4,4′-dmbipy^21^), can hardly be monitored by *in situ* IR spectroscopy, as the Mo dicarbonyl dimer readily converts to the tricarbonyl complex [Mo(6,6′-dmbipy)(CO)_3_Y]^−^, which is further reducible to [Mo(6,6′-dmbipy)(CO)_3_]^2–^. This assignment refines and rectifies the description in previous papers.^20,21^ The unprecedented thermal reactivity of [Mo(η^3^-2-R-allyl)(6,6′-dmbipy)(CO)_2_]_2_ precludes the cathodic recovery of [Mo(η^3^-2-R-allyl)(6,6′-dmbipy)(CO)_2_]^−^, having an inhibiting effect on the electrocatalytic reduction of CO_2_. The conversion of CO_2_ to CO in the early stages of the catalytic process facilitates the production of inactive [Mo(6,6′-dmbipy)(CO)_3_Y]^−^ replacing the catalyst and its dicarbonyl precursors. The mechanism of the peculiar formation of [Mo(6,6′-dmbipy)(CO)_3_Y]^−^ (also under argon) and determination of the ligand Y^–^ remain the targets of an ongoing study.

## Supporting Information Available

Complementary cyclic voltammograms, DFT calculations and the associated data tables, and additional spectroelectrochemical material (PDF)

## Accession Codes

CCDC 1989618, 1989622, and 1989623 contain the supplementary crystallographic data for this paper. These data can be obtained free of charge via www.ccdc.cam.ac.uk/data_request/cif, or by emailing data_request@ccdc.cam.ac.uk, or by contacting The Cambridge Crystallographic Data Centre, 12 Union Road, Cambridge CB2 1EZ, UK; fax: +44 1223 336033.

## Dedication

Dedicated to Professor Wolfgang Kaim on the occasion of his 70th birthday.
