# Supplementary material for: Effect of the 2-R-Allyl and Chloride Ligands on the Cathodic Paths of [Mo(η3-2-R-allyl)(α-diimine)(CO)2Cl] (R = H, CH3; α-diimine = 6,6′-Dimethyl-2,2′-bipyridine, Bis(p-tolylimino)acenaphthene)
Source: Organometallics. 2021 Jun 2;40(11):1598–613. doi: 10.1021/acs.organomet.1c00038 (PMC8289335; doi:10.1021/acs.organomet.1c00038)
Supplement: Supplementary file 1 — om1c00038_si_001.pdf [file om1c00038_si_001.pdf]

Supporting Information

Impact of the 2-R-Allyl and Chloride Ligands on the Cathodic Paths of [Mo(η^3-2-R-allyl)(α-diimine)$(CO)_2$Cl] (R = H, CH_3; α-diimine = 6,6′-dimethyl-2,2′-bipyridine and bis(*p*-tolylimino)acenaphthene)

James O. Taylor,[1] Ryan Culpeck,[1] Ann M. Chippindale,[1] Maria José Calhorda,[2] František Hartl[1,*]

[1] Department of Chemistry, University of Reading, Whiteknights, Reading, RG6 6DX, United Kingdom

[2] BioISI - Biosystems & Integrative Sciences Institute, Departamento de Química e Bioquímica, Faculdade de Ciências, Universidade de Lisboa, 1749-016 Lisbon, Portugal

Contents

	Title	Page
Table S1	Crystallographic data for complexes **1-3** (Chart 1 in the main text).	S3
Table S2	Selected bond lengths (Å) and angles (°) for [Mo(η^3-allyl))(6,6'-dmbipy)(CO)$_2$Cl].CH_2Cl_2 (**1**), [Mo(η^3-2-methallyl)(6,6'-dmbipy)(CO)$_2$Cl] (**2**) and [Mo(η^3-2-methallyl)(pTol-bian)(CO)$_2$Cl] (**3**).	S3
Table S3	DFT-calculated energies (kcal mol^{-1}) for **1**, **2** and **3** (= X), their cations [X]$^+$, their radical and derived 5-coordinate radicals [X-R], 5-coordinate anions [X-A]$^-$ (obtained by the loss of chloride), 6-coordinate [X-PrCN]$^-$ anions, and dimers [X-D] (for the molecular structures, see Scheme 1).	S4
Table S4	Relevant DFT-calculated distances (Å) in **1**, **2** and **3** (= X), as well as their cations, radical anions [X]$^{\bullet-}$ and derived 5-coordinate radicals [X-R] and reduced anions [X-A]$^-$ (obtained by the loss of chloride).	S5
Table S5	DFT-calculated wavenumbers of the symmetric and antisymmetric CO-stretching modes (cm^{-1}) in **1-3** (= X) and their reduction and oxidation products (see Scheme 1 in the main text).	S6
Figure S1	Cyclic voltammograms of complex **1** at (a) T = 298 K and (b) 195 Kin PrCN/Bu_4NPF_6. The arrow indicates the initial scan direction. Conditions: Pt microdisc electrode, v = 100 mV s^{-1}.	S6
Figure S2	Cyclic voltammograms of complex **2** at (a) T = 298 K and (b) 195 K in PrCN/Bu_4NPF_6. The arrow indicates the initial scan direction. Conditions: Pt microdisc electrode, v = 100 mV s^{-1}.	S7
Figure S3	Cyclic voltammograms of complex **3** at (a) T = 298 K and (b) 195 K in PrCN/Bu_4NPF_6. The arrow indicates the initial scan direction. Conditions: Pt microdisc electrode, v = 100 mV s^{-1}.	S7
Figure S4	DFT-optimized structures, from top to bottom of: the parent complex, [Mo(η^3-allyl)(6,6′-dmbipy)(CO)$_2$Cl] (**1**) (the equatorial and axial isomers); cation [**1**]$^+$; 1e$^-$ reduced radical anion [**1**]$^{\bullet-}$ (the equatorial and axial isomers); dimer [**1**-D]; 5-coordinate SP radical [**1**-R], 2e$^-$ reduced 5-coordinate anion [**1**-A]$^-$, and CO_2 complex [**1**-CO_2]$^-$, with the relevant bond lengths (Å).	S8
Figure S5	DFT-optimized structure, from top to bottom, of: the parent complex [Mo(η^3-2-methallyl)(pTol-bian)(CO)$_2$Cl] (**3**) (the equatorial and axial isomers), and cation [**3**]$^+$; 1e$^-$ reduced radical anion [**3**]$^{\bullet-}$ (the equatorial and axial isomers) and the PrCN anionic complex [**3**-PrCN]$^-$; 5-coordinate radical [**3**-R] and 2e$^-$ reduced 5-coordinate anion [**3**-A]$^-$, with the relevant bond lengths (Å).	S9
Figure S6	Frontier orbitals of the parent complex [Mo(η^3-allyl)(6,6′-dmbipy)(CO)$_2$Cl] (**1**). Energies (eV): HOMO (H) -4.91, LUMO (H) -3.61.	S10
Figure S7	Frontier orbitals of the parent complex [Mo(η^3-2-methallyl)(pTol-bian)(CO)$_2$Cl] (**3**). Energies (eV): HOMO (H) -4.91, LUMO (H) -3.85.	S10
Figure S8	Frontier orbitals of the 5-coordinate anion [Mo(η^3-allyl)(6,6′-dmbipy)(CO)$_2$]$^-$, [**1**-A]$^-$. Energies (eV): HOMO (H) -2.86, LUMO (H) -1.61.	S11
Figure S9	Frontier orbitals of the 5-coordinate anion [Mo(η^3-2-methallyl)(pTol-bian)(CO)$_2$]$^-$, [**3**-A]. Energies (eV): HOMO (H) -3.38, LUMO (H) -2.25.	S11

Figure S10 IR SEC monitoring of (a) the 1e⁻ oxidation of [Mo(η^3-allyl)(6,6′-dmbipy)$(CO)_2$Cl] (**1**) (↓) to slowly decomposing cation [**1**]$^+$ (↑ ↓), and (b) the 1e⁻ oxidation of **2** (↓) to stable [**2**]$^+$ (↑). Conditions: an OTTLE cell, THF/Bu_4NPF_6, T = 298 K. S12

Figure S11 IR SEC monitoring of the overall 2e⁻ reduction of [Mo(η^3-2-methallyl)(6,6′- dmbipy)$(CO)_2$Cl], **2** (↓) at R1 to the 5-coordinate anion, [**2**-A]⁻ (↑). Conditions: a cryostatted OTTLE cell, THF/Bu_4NPF_6, T = 255 K. S12

Figure S12 Complete cyclic voltammograms of complexes **1-3** including the anodic traces in THF/Bu_4NPF_6. (a) **1**, T = 298 K. (b) **1**, T = 195 K. (c) **2**, T = 298 K. (d) **2**, T = 195 K. (e) **3**, T = 298 K, (f) **3**, T = 195 K. The asterisk (*) indicates the internal ferrocene standard; the symbol + indicates the auxiliary decamethyl ferrocene standard. S13

Figure S13 Complete cyclic voltammograms of complexes **1-3** including the anodic traces in PrCN/Bu_4NPF_6. (a) **1**, T = 298 K. (b) **1**, T = 195 K. (c) **2**, T = 298 K. (d) **2**, T = 195 K. (e) **3**, T = 298 K, (f) **3**, T = 195 K. The asterisk (*) indicates the internal ferrocene standard. S14

Table S1. Crystallographic data for complexes 1-3 (Chart 1 in the main text).

Complex	**1**	**2**	**3**
Formula	$C_{17}H_{17}ClMoN_2O_2$. (CH_2Cl_2)	$C_{18}H_{19}ClMoN_2O_2$	$C_{32}H_{27}ClMoN_2O_2$
M_r	497.64 (412.73)[a]	426.75	602.97
Crystal System	monoclinic	monoclinic	triclinic
Space Group	$P2_1/n$	$P2_1/m$	P-1
Z	4	2	2
a / Å	10.56215(1)	8.03542(1)	9.82412(2)
b / Å	10.53608(1)	11.43157(2)	12.11344(2)
c / Å	17.44881(2)	9.60033(2)	12.88898(2)
α / °	90	90	74.449(3)
β / °	90.256(2)	93.890(3)	70.667(3)
γ / °	90	90	68.437(4)
V / Å^3	1941.748(4)	879.830(4)	1327.19(4)
ρ_{calc} /g cm^{-3}	1.702	1.611	1.509
Radiation	Cu Kα	Cu Kα	Cu Kα
T / K	100	200	100
Crystal habit	Red Plate	Yellow Plate	Orange Plate
Crystal dimensions / mm	0.05 × 0.096 × 0.161	0.034 × 0.106 × 0.186	0.050 × 0.086 × 0.142
μ /mm^{-1}	9.456	7.598	5.227
$R(F)$, $R_w(F)$	0.0326, 0.0435	0.0437, 0.0595	0.0349, 0.0532
CCDC code	1989618	1989622	1989623

[a] Excludes solvent.

Table S2. Selected bond lengths (Å) and angles (°) for [Mo(η^3-allyl))(6,6'-dmbipy)(CO)$_2$Cl].CH$_2$Cl$_2$ (1), [Mo(η^3-2-methallyl)(6,6'-dmbipy)(CO)$_2$Cl] (2) and [Mo(η^3-2-methallyl)(pTol-bian)(CO)$_2$Cl] (3).[a]

Complex	**1**	**2**	**3**
Mo(1)–Cl(1)	2.4914(8)	2.5145(15)	2.4873(7)
Mo(1)–N(1)	2.300(3)	2.283(4)	2.261(2)
Mo(1)–N(2)	2.292(3)		2.233(2)
Mo(1)–C(1)	1.957(3)	1.958(5)	1.984(3)
Mo(1)–C(2)	1.956(3)	2.324(5)	1.972(3)
Mo(1)–C(3)	2.325(3)	2.221(8)	2.318(3)
Mo(1)–C(4)	2.211(3)		2.258(3)
Mo(1)–C(5)	2.338(3)		2.334(3)
C(2)–O(1)	1.157(4)	1.152(6)	1.139(4)
C(3)–O(2)	1.155(3)		1.145(4)
N(1)–Mo(1)–N(2)	72.30(10)	73.31(19)	73.68(8)
C(1)–Mo(1)–C(2)	76.06(14)	74.3(3) [b]	80.64(14)

[a] The numbering scheme corresponds to that of the experimental structure of **1** (Figure 1 in the main text). [b] Corresponds to C(1)–Mo(1)–C(1)'.

Table S3. DFT-calculated energies (kcal mol^{-1}) for 1, 2, and 3 (= X), their cations [X]$^+$, their radical and derived 5-coordinate radicals [X-R], 5-coordinate anions [X-A]$^-$ (obtained by the loss of chloride), 6-coordinate [X-PrCN]$^-$ anions, and dimers [X-D] (for the molecular structures, see Scheme 1).

Complex	**1** / kcal mol^{-1}	**2** / kcal mol^{-1}	**3** / kcal mol^{-1}
**X** eq	-5912.02	-6286.30	-9800.54
**X** ax	-5906.79	-6278.38	-9798.82
[**X**]$^+$ eq	-5792.09	-6168.36	-9682.62
[**X**]$^{\bullet-}$ eq	-5975.47	-6348.98	-9877.25
[**X**]$^{\bullet-}$ ax	-5969.64	-6339.98	-9874.86
[**X**-R] SP	-5814.16	-6190.84	-9717.81
[**X**-R] TBP	-5812.60	Also SP	-[a]
[**X**-A]$^-$ diamag.	-5883.55	-6259.58	-9798.24
[**X**-A]$^-$ paramag.	-5871.71	-6240.76	-9777.82

[**X**-PrCN]⁻	-7.467.20	-7832.42	-11384.38
[**X**-D]	-11632.00	-12383.32	-[a]

[a] Not converged.

Table S4. Relevant DFT-calculated distances (Å) in 1, 2, and 3 (= X), as well as their cations, radical anions [X]•⁻ and derived 5-coordinate radicals [X-R] and reduced anions [X-A]⁻ (obtained by the loss of chloride).[a]

Bond		**X**		[**X**]•⁻		[**X**]⁺	[**X**-R]	[**X**-A]⁻
	Exp(eq)	equat	axial	equat	axial	equat	SP	SP

Complex **1**								
Mo–C(3)	2.325	2.356	2.359	2.346	2.353	2.380	2.325	2.326
Mo–C(4)	2.211	2.237	2.234	2.225	2.235	2.312	2.200	2.211
Mo–C(5)	2.338	2.354	2.341	2.450	2.339	2.381	2.326	2.329
Mo–C(1)(O)	1.957	1.953	1.943	1.955	1.948	2.020	1.954	1.961
Mo–C(2)(O)	1.956	1.952	1.944	1.954	1.936	2.020	1.954	1.961
Mo–N(1) (trans CO)	2.300	2.328	2.371	2.290	2.325	2.295	2.235	2.185
Mo–N(2)	2.292	2.324	2.281	2.286	2.256	2.292	2.236	2.187
C(11)–C(12) [b]	1.482	1.481	1.478	1.433	1.428	1.479	1.454	1.421
Mo–Cl(1)	2.491	2.510	2.177	2.560	2.256	2.398	-	-
Complex **2**								
Mo–C(3)	2.324	2.354	2.407	2.342	2.337	2.369	2.326	2.327
Mo–C(4)	2.221	2.287	2.314	2.265	2.311	2.387	2.224	2.327
Mo–C(5)	2.324	2.353	2.334	2.341	2.392	2.370	2.327	2.327
Mo–C(1)(O)	1.958	1.952	1.925	1.953	1.934	2.017	1.949	1.957
Mo–C(2)(O)	1.958	1.952	1.940	1.952	1.930	2.017	1.950	1.958
Mo–N(1) (trans CO)	2.283	2.319	2.345	2.283	2.314	2.289	2.238	2.186
Mo–N(2)	2.283	2.318	2.287	2.281	2.264	2.290	2.241	2.189
C(5)–C(5’) [b]	1.488	1.481	1.475	1.433	1.431	1.480	1.452	1.420
C_{meso}–CH_3 (2-methallyl)	1.539	1.508	1.509	1.508	1.510	1.503	1.511	1.511
Mo–Cl(1)	2.515	2.517	2.582	2.143	2.624	2.406	-	-
Complex **3**								
Mo–C(3)	2.318	2.354	2.356	2.334	2.357	2.351	2.327	2.332
Mo–C(4)	2.258	2.303	2.285	2.254	2.274	2.367	2.198	2.211
Mo–C(5)	2.334	2.353	2.353	2.333	2.349	2.350	2.325	2.330
Mo–C(1)(O)	1.984	1.973	1.952	1.956	1.935	2.033	1.943	1.954
Mo–C(2)(O)	1.972	1.972	1.967	1.955	1.950	2.033	1.945	1.954
Mo–N(1) (trans CO)	2.261	2.259	2.309	2.276	2.320	1.153	2.201	2.160
Mo–N(2)	2.333	2.253	2.220	2.270	2.214	1.153	2.220	2.153
C(14)–C(18) [b]	1.498	1.481	1.476	1.442	1.436	1.496	1.447	1.403
C_{meso}–CH_3 (2-methallyl)	1.507	1.507	1.507	1.508	1.509	1.503	1.512	1.513
Mo–Cl(1)	2.487	2.510	2.579	2.572	2.652	2.425	-	-

[a] The numbering schemes correspond to that of the experimental structure of **1** (Figure 1 in the main text), with the exception of the footnote b. [b] The inter-ring C–C bond in 6,6'-dmbipy and the corresponding C–C bond in the five-membered metallacycle in **3**.

Table S5. DFT-calculated wavenumbers of the symmetric and antisymmetric CO-stretching modes (cm^{-1}) in 1-3 (= X) and their reduction and oxidation products (see Scheme 1 in the main text).

Complex	X	$[X]^{\bullet-}$		$[X]^{+}$	[X-R]		$[X-A]^{-}$		[X-D] [a]	$[X-CO_2]^{-}$	$[X-PrCN]^{-}$
	equat	equat	axial	equat	SP	TBP	TBP	SP	SP	equat	axial
**1**											
v_s(C≡O)	1878	1852	1847	1983	1851	1830	-	1804	1855, 1847	1829	1817[b]
v_a(C≡O)	1797	1759	1752	1925	1759	1743	-	1702	1782	1741	1715
**2**											
v_s(C≡O)	1879	1851	1844	1979	1857		-	1802	1855, 1847	1830	1827
v_a(C≡O)	1797	1760	1750	1920	1759		-	1701	1782	1742	1742
**3**											
v_s(C≡O)	1891	1858	1856	1987	1868		-	1827	-	-	1816
v_a(C≡O)	1821	1758	1769	1939	1781		-	1734	-	-	1729

[a] Calculated three strong v(CO) modes of the genuine Mo–Mo dimer. The fourth v(CO) absorption is very weak. [b] Equatorial isomer.

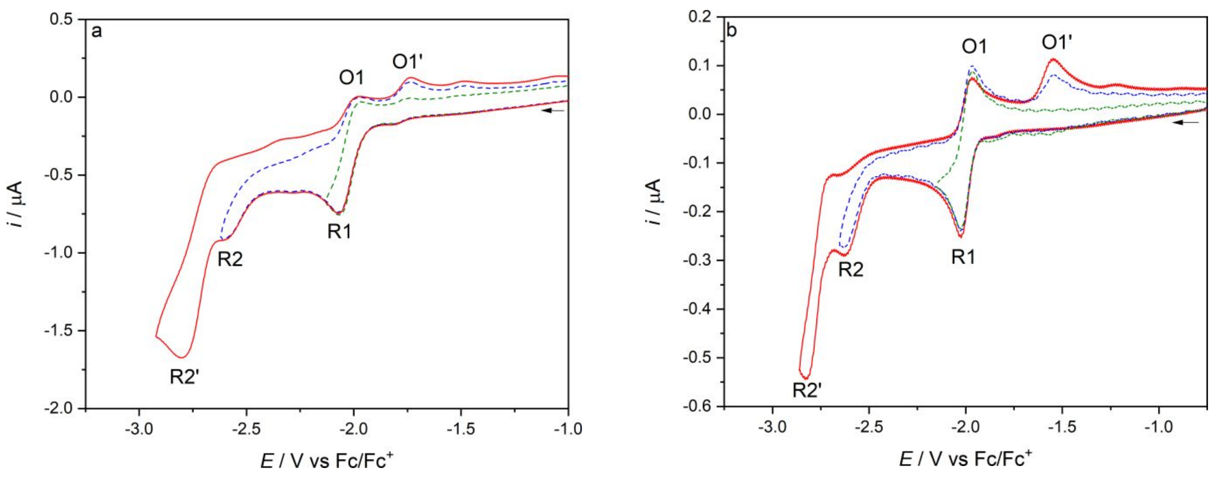

Figure S1. Cyclic voltammograms of complex **1** at (a) T = 298 K and (b) 195 K in PrCN/Bu_4NPF_6. The arrow indicates the initial scan direction. Conditions: Pt microdisc electrode, v = 100 mV s^{-1}.

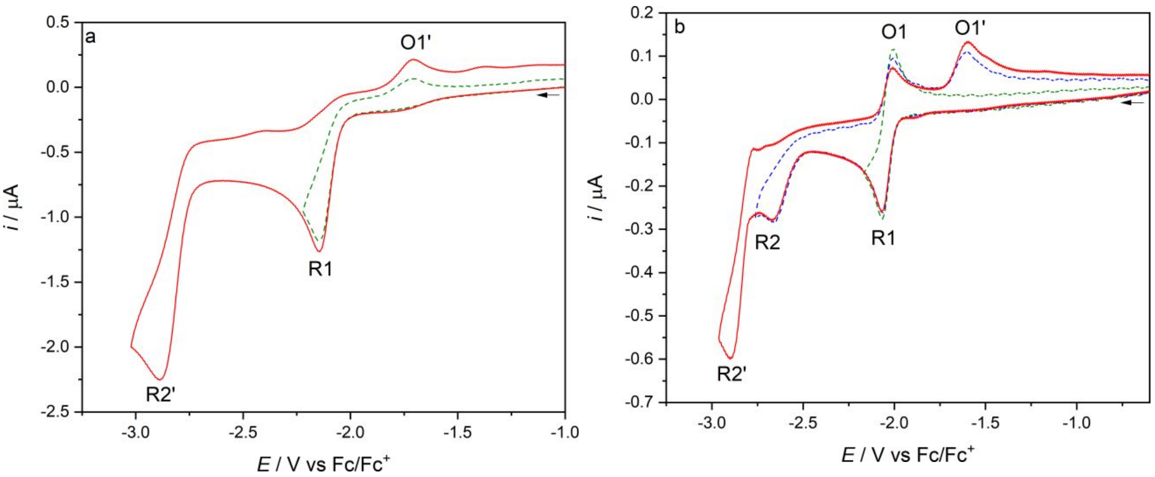

Figure S2. Cyclic voltammograms of complex **2** at (a) T = 298 K and (b) 195 K in PrCN/Bu_4NPF_6. The arrow indicates the initial scan direction. Conditions: Pt microdisc electrode, v = 100 mV s^{-1}.

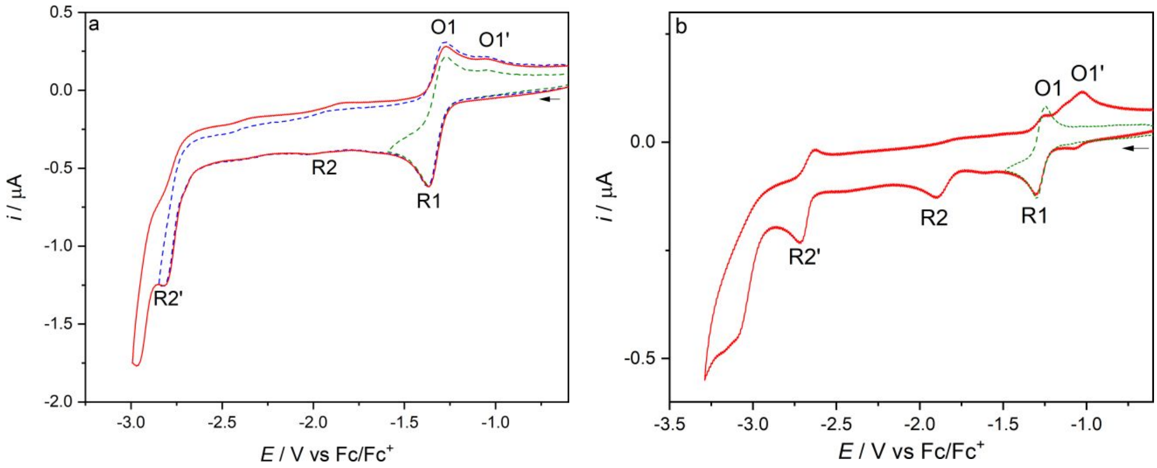

Figure S3. Cyclic voltammograms of complex **3** at (a) T = 298 K and (b) 195 K in PrCN/Bu_4NPF_6. The arrow indicates the initial scan direction. Conditions: Pt microdisc electrode, v = 100 mV s^{-1}.

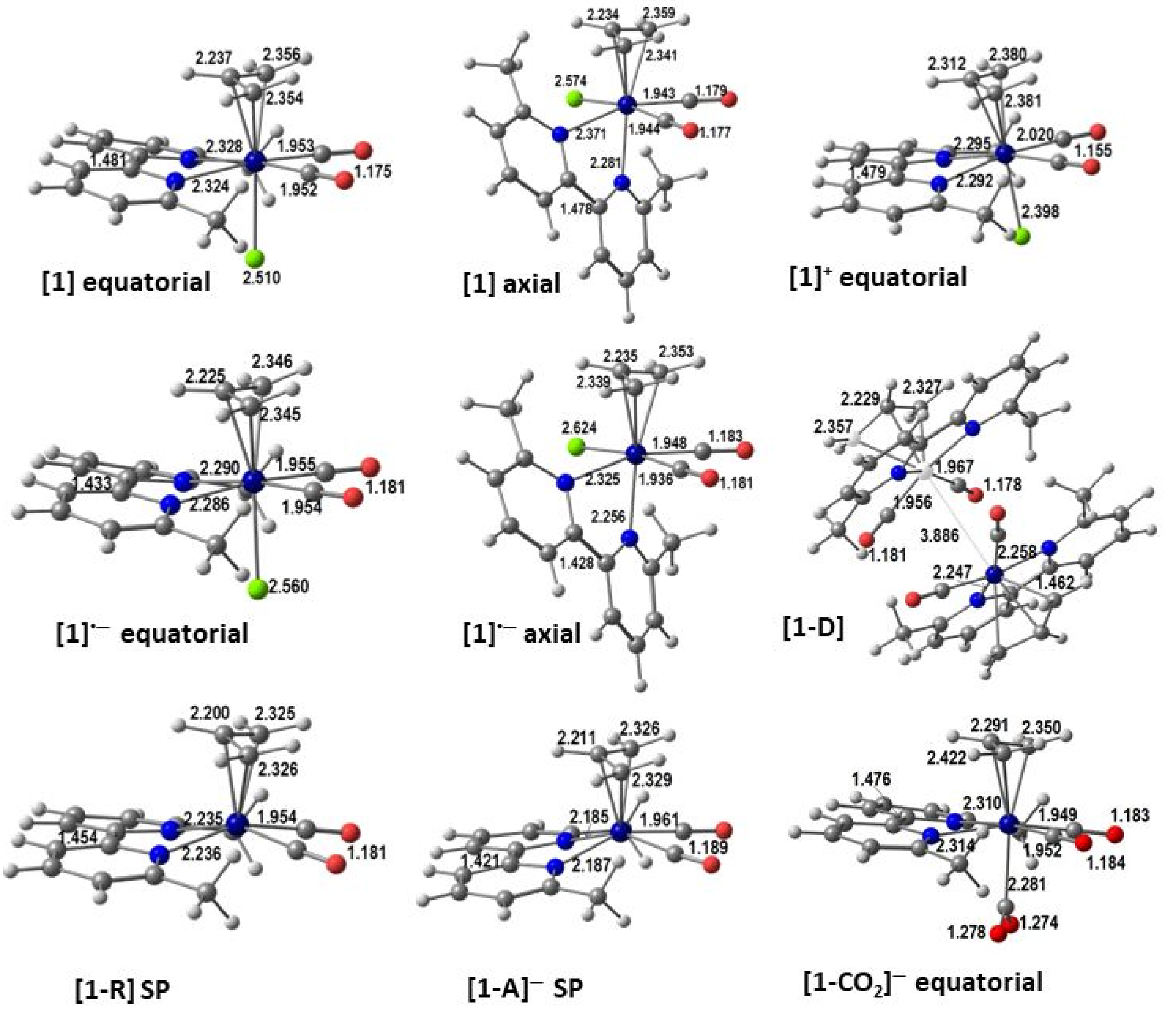

Figure S4. DFT-optimized structures, from top to bottom of: the parent complex, [Mo(η^3-allyl)(6,6′-dmbipy)$(CO)_2$Cl] (**1**) (the equatorial and axial isomers); cation [**1**]$^+$; 1e$^-$ reduced radical anion [**1**]$^{\bullet-}$ (the equatorial and axial isomers); dimer **[1-D]**; 5-coordinate SP radical [**1**-R], 2e$^-$ reduced 5-coordinate anion [**1**-A]$^-$, and CO_2 complex [**1**-CO_2]$^-$**,** with the relevant bond lengths (Å).

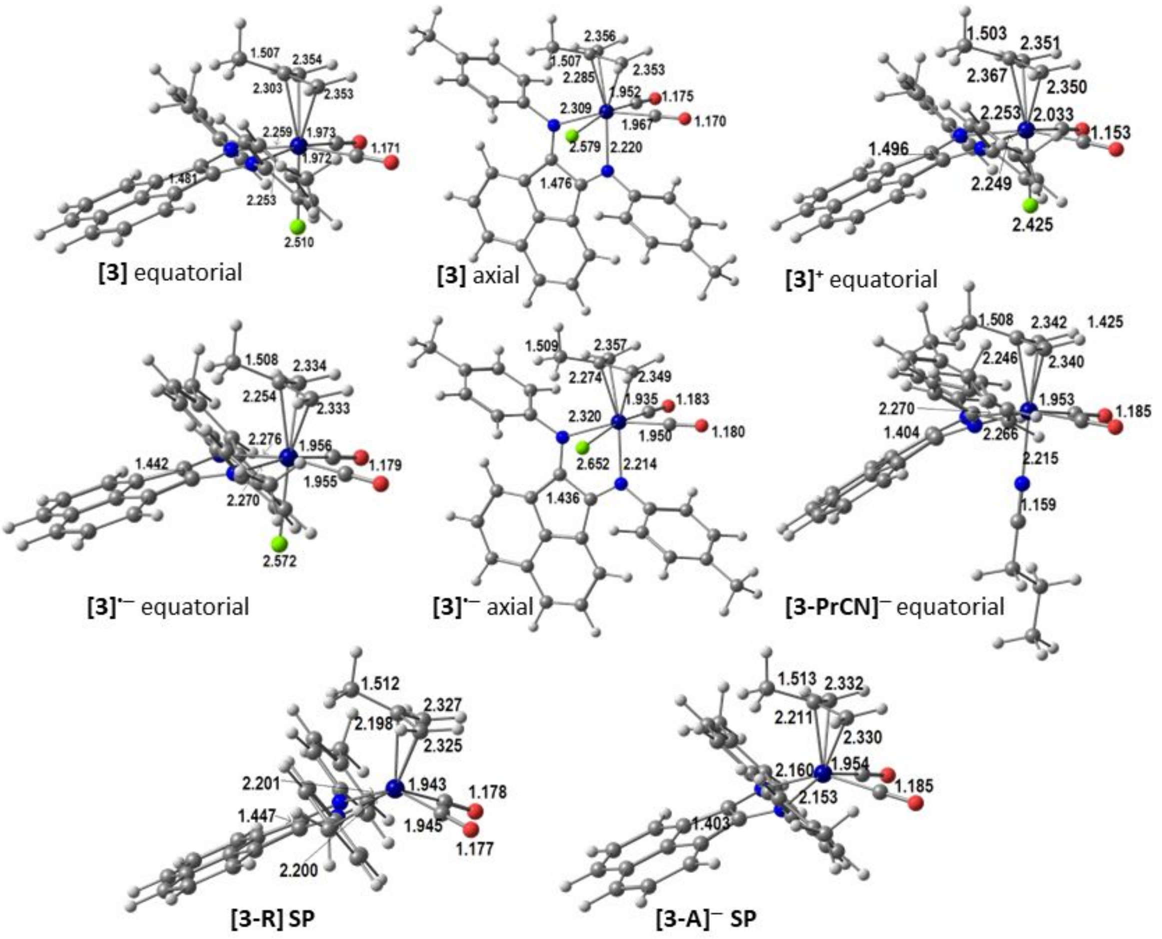

Figure S5. DFT-optimized structure, from top to bottom, of: the parent complex [Mo(η^3-2-methallyl)(pTol-bian)(CO)$_2$Cl] (**3**) (the equatorial and axial isomers), and cation [**3**]$^+$; 1e$^-$ reduced radical anion [**3**]$^{\bullet-}$ (the equatorial and axial isomers) and the PrCN anionic complex [**3**-PrCN]$^-$; 5-coordinate radical [**3**-R] and 2e$^-$ reduced 5-coordinate anion [**3**-A]$^-$, with the relevant bond lengths (Å).

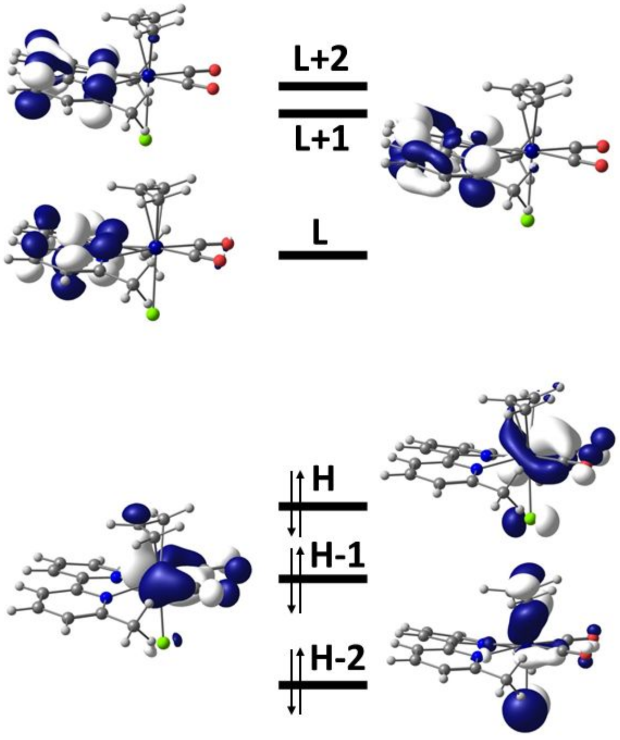

Figure S6. Frontier orbitals of the parent complex [Mo(η^3-allyl)(6,6′-dmbipy)$(CO)_2$Cl] (**1**). Energies (eV): HOMO (H) -4.91, LUMO (H) -3.61.

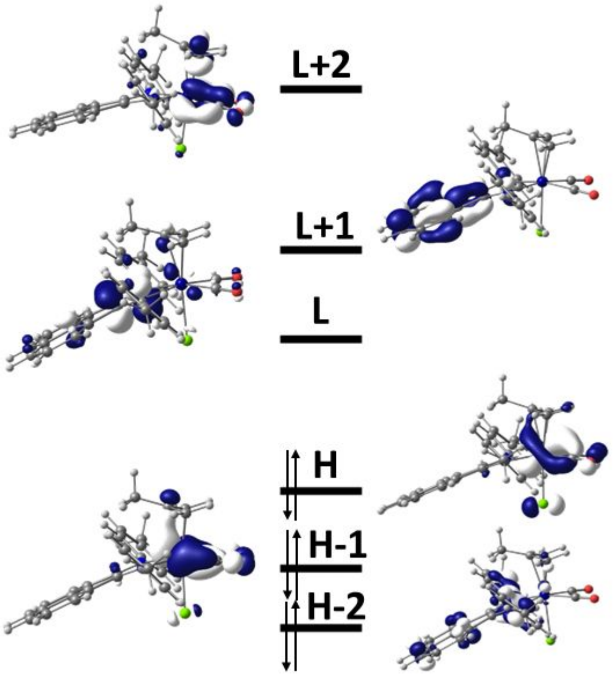

Figure S7. Frontier orbitals of the parent complex [Mo(η^3-2-methallyl)(pTol-bian)$(CO)_2$Cl] (**3**). Energies (eV): HOMO (H) -4.91, LUMO (H) -3.85.

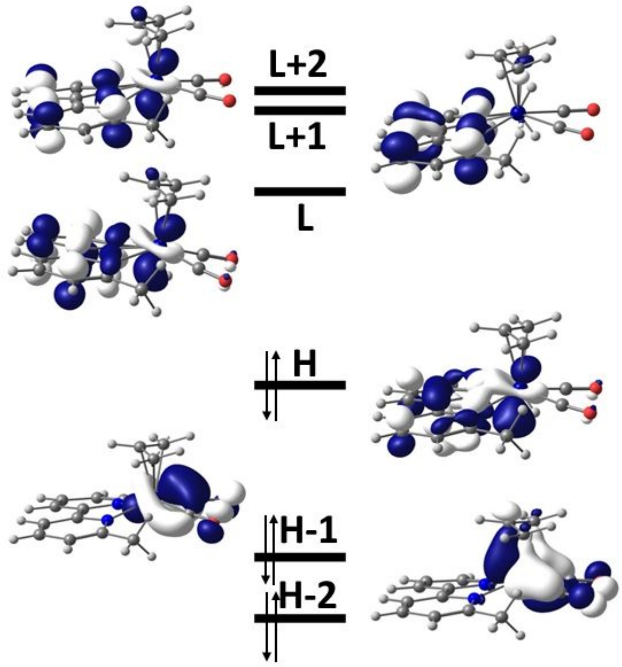

Figure S8. Frontier orbitals of the 5-coordinate anion [Mo(η^3-allyl)(6,6′-dmbipy)(CO)$_2$]$^-$, [**1**-A]$^-$. Energies (eV): HOMO (H) -2.86, LUMO (H) -1.61.

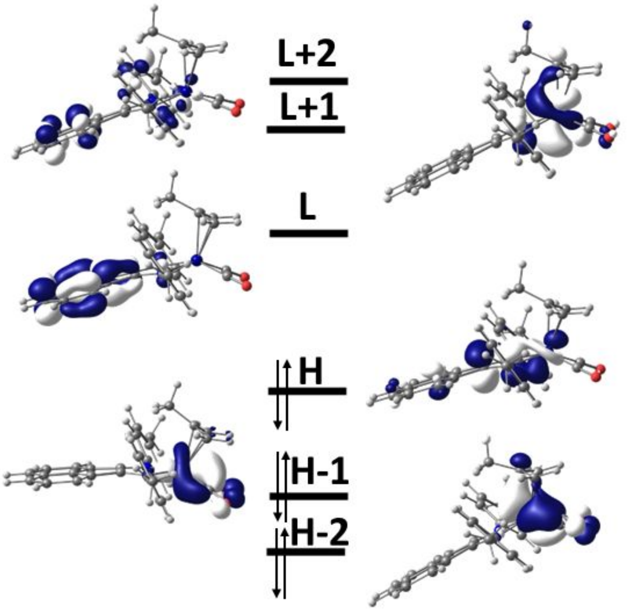

Figure S9. Frontier orbitals of the 5-coordinate anion [Mo(η^3-2-methallyl)(pTol-bian)(CO)$_2$]$^-$, [**3**-A]$^-$. Energies (eV): HOMO (H) -3.38, LUMO (H) -2.25.

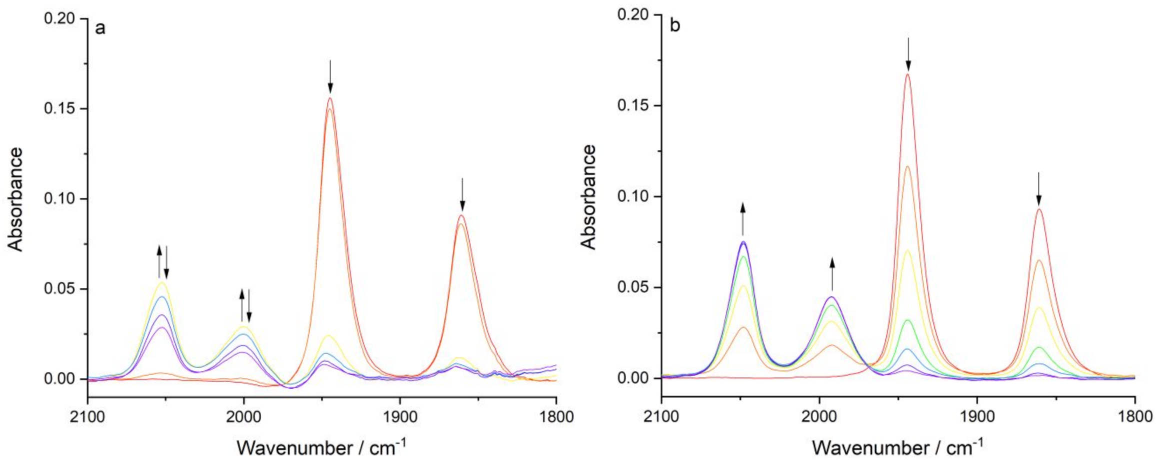

Figure S10. IR SEC monitoring of (a) the 1e⁻ oxidation of [Mo(η^3-allyl)(6,6′-dmbipy)$(CO)_2$Cl] (**1**) (↓) to slowly decomposing cation [**1**]$^+$ (↑ ↓), and (b) the 1e⁻ oxidation of **2** (↓) to stable [**2**]$^+$ (↑). Conditions: an OTTLE cell, THF/Bu_4NPF_6, *T* = 298 K.

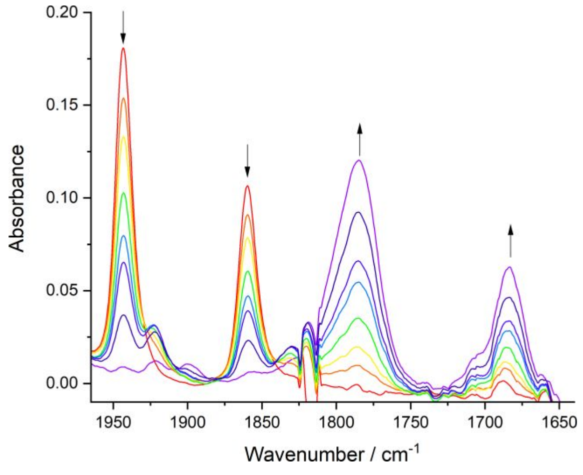

Figure S11. IR SEC monitoring of the overall 2e⁻ reduction of [Mo(η^3-2-methallyl)(6,6′- dmbipy)$(CO)_2$Cl], **2** (↓) at R1 to the 5-coordinate anion, [**2**-A]⁻ (↑). Conditions: a cryostatted OTTLE cell, THF/Bu_4NPF_6, *T* = 255 K.

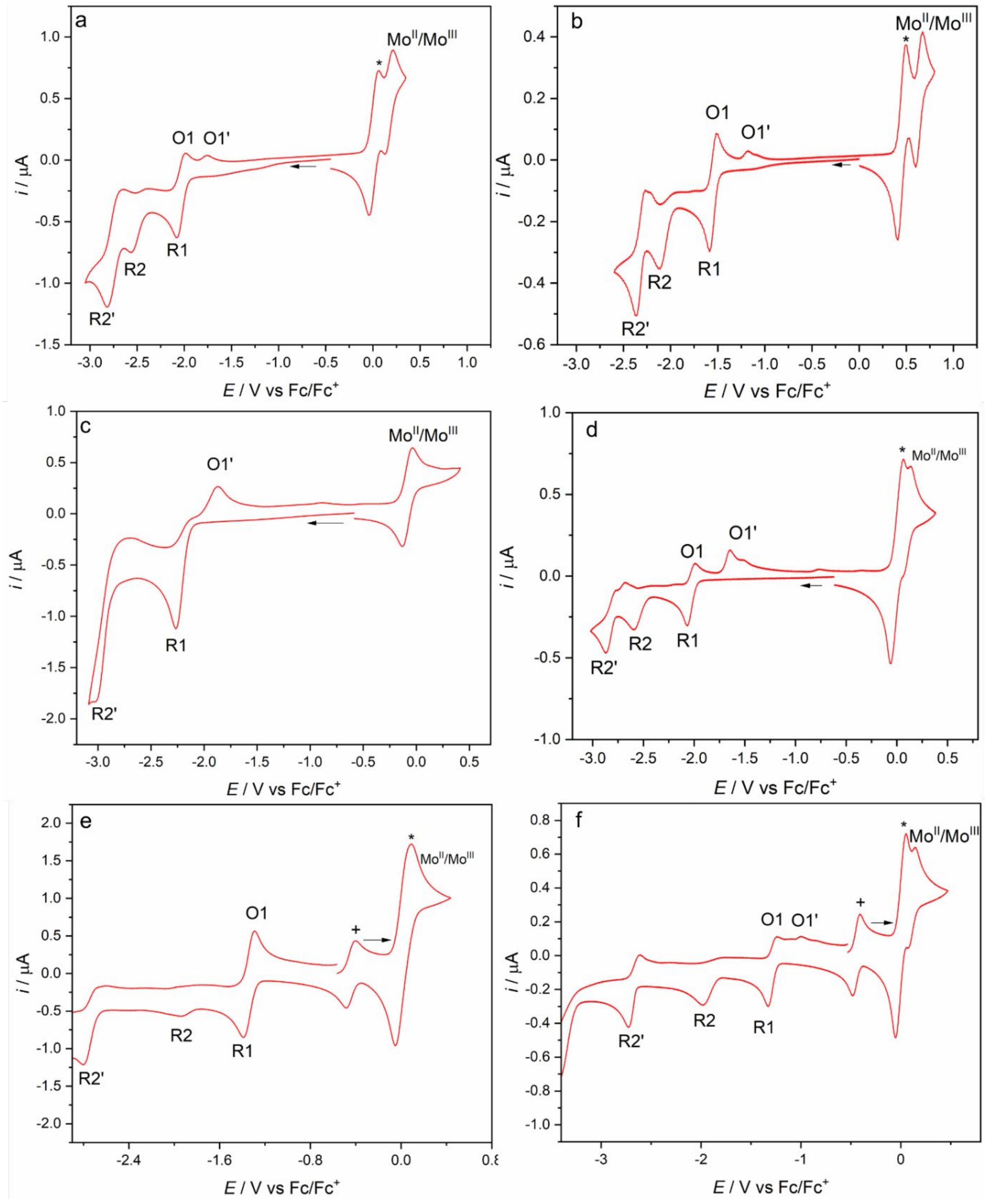

Figure S12. Complete cyclic voltammograms of complexes **1**-**3** including the anodic traces in THF/Bu_4NPF_6. (a) **1**, T = 298 K. (b) **1**, T = 195 K. (c) **2**, T = 298 K. (d) **2**, T = 195 K. (e) **3**, T = 298 K, (f) **3**, T = 195 K. The asterisk (*) indicates the internal ferrocene standard; the symbol + indicates the auxiliary decamethyl ferrocene standard.

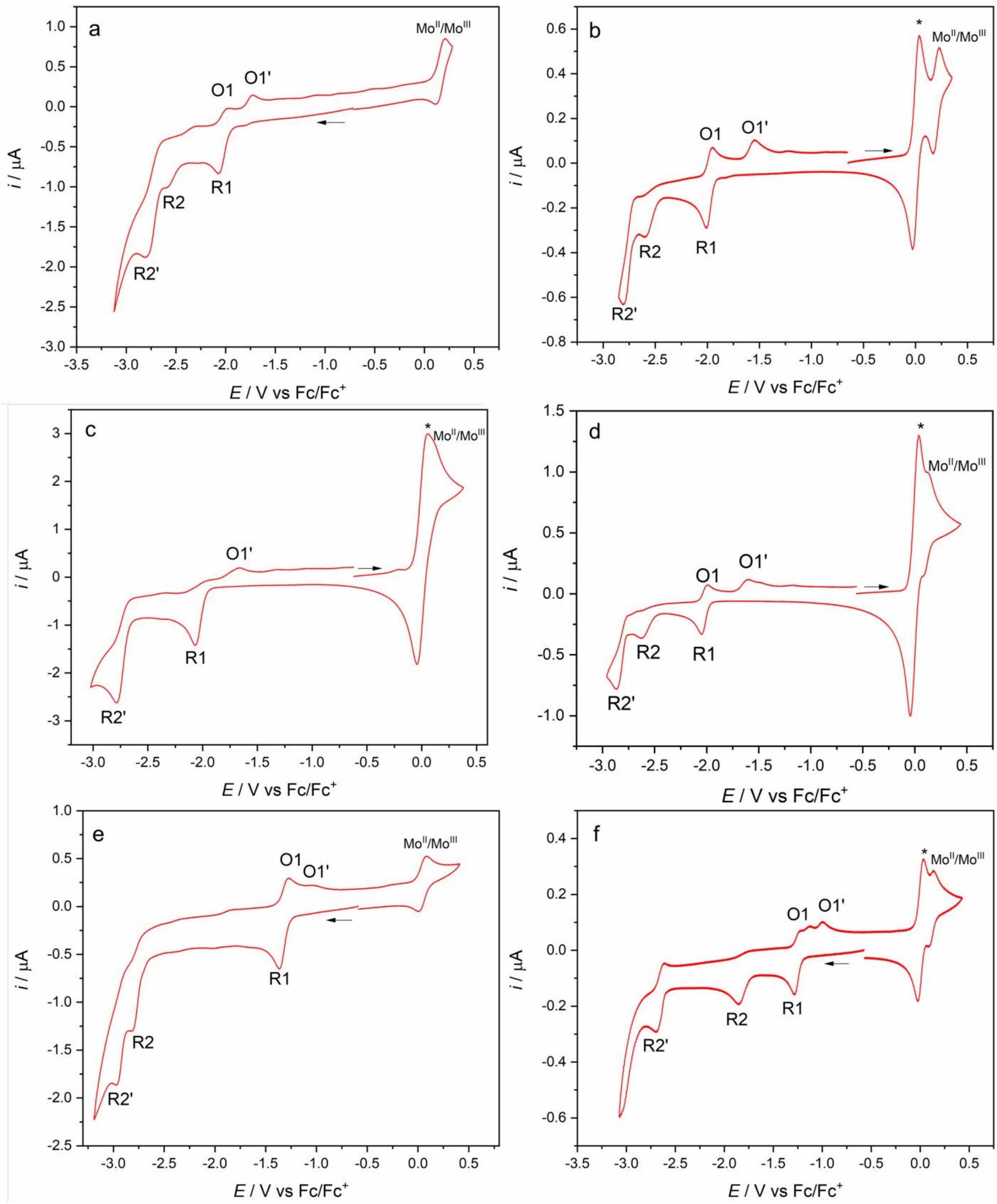

Figure S13. Complete cyclic voltammograms of complexes **1-3** including the anodic traces in PrCN/Bu_4NPF_6. (a) **1**, *T* = 298 K. (b) **1**, *T* = 195 K. (c) **2**, *T* = 298 K. (d) **2**, *T* = 195 K. (e) **3**, *T* = 298 K, (f) **3**, *T* = 195 K. The asterisk (*) indicates the internal ferrocene standard.
